# Monoterpene production by the carotenogenic yeast *Rhodosporidium toruloides*

**DOI:** 10.1186/s12934-019-1099-8

**Published:** 2019-03-18

**Authors:** Xun Zhuang, Oliver Kilian, Eric Monroe, Masakazu Ito, Mary Bao Tran-Gymfi, Fang Liu, Ryan W. Davis, Mona Mirsiaghi, Eric Sundstrom, Todd Pray, Jeffrey M. Skerker, Anthe George, John M. Gladden

**Affiliations:** 1 Biomass Science and Conversion Technology, Sandia National Laboratories, 7011 East Ave, Livermore, CA 94551 USA; 2 Advanced Biofuels Process Development Unit (ABPDU), Lawrence Berkeley National Laboratory, 5885 Hollis St, Emeryville, CA 94608 USA; 3 Energy Bioscience Institute, 2151 Berkeley Way, Berkeley, CA 94704 USA; 4 Lawrence Berkeley National Laboratory, 1 Cyclotron Rd, Berkeley, CA 94720 USA; 5 Deconstruction Division, Joint BioEnergy Institute/Sandia National Laboratories, 5885 Hollis St, Emeryville, CA 94608 USA

**Keywords:** *Rhodosporidium toruloides*, Monoterpene, 1,8-Cineole, Biofuel

## Abstract

**Background:**

Due to their high energy density and compatible physical properties, several monoterpenes have been investigated as potential renewable transportation fuels, either as blendstocks with petroleum or as drop-in replacements for use in vehicles (both heavy and light-weight) or in aviation. Sustainable microbial production of these biofuels requires the ability to utilize cheap and readily available feedstocks such as lignocellulosic biomass, which can be depolymerized into fermentable carbon sources such as glucose and xylose. However, common microbial production platforms such as the yeast *Saccharomyces cerevisiae* are not naturally capable of utilizing xylose, hence requiring extensive strain engineering and optimization to efficiently utilize lignocellulosic feedstocks. In contrast, the oleaginous red yeast *Rhodosporidium toruloides* is capable of efficiently metabolizing both xylose and glucose, suggesting that it may be a suitable host for the production of lignocellulosic bioproducts. In addition, *R. toruloides* naturally produces several carotenoids (C40 terpenoids), indicating that it may have a naturally high carbon flux through its mevalonate (MVA) pathway, providing pools of intermediates for the production of a wide range of heterologous terpene-based biofuels and bioproducts from lignocellulose.

**Results:**

Sixteen terpene synthases (TS) originating from plants, bacteria and fungi were evaluated for their ability to produce a total of nine different monoterpenes in *R. toruloides*. Eight of these TS were functional and produced several different monoterpenes, either as individual compounds or as mixtures, with 1,8-cineole, sabinene, ocimene, pinene, limonene, and carene being produced at the highest levels. The 1,8-cineole synthase *HYP3* from *Hypoxylon* sp. *E74060B* produced the highest titer of 14.94 ± 1.84 mg/L 1,8-cineole in YPD medium and was selected for further optimization and fuel properties study. Production of 1,8-cineole from lignocellulose was also demonstrated in a 2L batch fermentation, and cineole production titers reached 34.6 mg/L in DMR-EH (Deacetylated, Mechanically Refined, Enzymatically Hydorlized) hydrolysate. Finally, the fuel properties of 1,8-cineole were examined, and indicate that it may be a suitable petroleum blend stock or drop-in replacement fuel for spark ignition engines.

**Conclusion:**

Our results demonstrate that *Rhodosporidium toruloides* is a suitable microbial platform for the production of non-native monoterpenes with biofuel applications from lignocellulosic biomass.

**Electronic supplementary material:**

The online version of this article (10.1186/s12934-019-1099-8) contains supplementary material, which is available to authorized users.

## Background

Renewable energy technologies have been gaining increasing public and scientific interest due to increasing global energy demands, fast depletion of fossil fuels, and an expanded awareness of issues arising from petroleum-derived greenhouse gas emissions [1–3]. The global transportation sector has high energy demands and is dependent on the availability of high energy density liquid fuels for use in light/heavy-duty vehicles, and in aviation. In the United States, gasoline is the dominant transportation fuel consumed, and therefore development of renewable gasoline alternatives could have a large positive impact on addressing the aforementioned issues. In fact, the federal program Renewable Fuel Standard (RFS), which requires transportation fuel sold in the US to contain a minimum volume of renewable fuel, has helped promote the development of these alternatives [4]. However, 80% of today’s transportation fuels are still derived from petroleum, so there is still plenty of opportunity to further develop renewable transportation fuels. Lignocellulosic biomass has been identified as an abundant carbon source for the production of biofuels, and has been the subject of intensive investigation in the past decades, resulting in the development of a number of bio-based gasoline, diesel and jet fuel alternatives. The 2016 DOE Billion-Ton study reported that the US could produce at least 1 billion dry tons of lignocellulosic biomass annually by 2040, suggesting that developing lignocellulosic-derived biofuels could play a major role in the domestic production of renewable transportation fuels [5].

Currently, the majority of vehicles driven in the US use two types of internal combustion engines: spark ignition (SI) and compression ignition (CI) engines. Corn grain-based ethanol and soy bean oil-derived biodiesel are currently the two predominantly used renewable transportation fuels for these engines. In 2014, bioethanol production reached 14.1 billion gallons [5]. However, although ethanol provides a performance enhancement to conventional gasoline by boosting its octane number, there are several aspects of ethanol that make it a less-than-ideal biofuel. For example, it contains only 70% of the energy density of gasoline, and its corrosivity and hygroscopicity make it incompatible with the existing transportation petroleum fuel distribution infrastructure [2, 3]. Ideally, the properties of a biofuel would more closely match or exceed those of conventional petroleum-based fuels. So, while ethanol is a good first step toward adoption of renewable transportation fuels, there is a need to develop biofuels with better fuel properties, such as increased energy density, increased compatibility with current distribution systems and engines, and decreased toxicity of emissions.

Terpenes and terpenoids are a large and diverse family of natural products with a number of potential industrial applications, including pharmaceuticals, flavors, fragrances, and most recently as energy-dense advanced biofuels [6–8]. Physical properties of several terpenes such as low viscosities, flash and freezing points, high energy densities, and high volumetric net heats of combustion (NHOC) [8], indicate that they would be good biofuel candidates. For example, monoterpenes including pinene, sabinene, and terpinene are currently being explored as jet fuel replacement [3, 9–11]. In addition, hydrogenated limonene and β-pinene have been shown to be suitable as diesel additives [8]. Finally, the monoterpenoid eucalyptol or 1,8-cineole may have use in both SI and CI engines [12–16].

Due to their numerous potential industrial applications, many terpenes have been produced heterologously in *E. coli* and *S. cerevisiae* microbes. For example, the sesquiterpenes amorphadiene and artemisinic acid, both precursors to the antimalarial drug artemisinin have been engineered into both *E. coli* and *S. cerevisiae* [17–26]. Compared to sesquiterpenes, monoterpenes are more challenging to produce, typically being made in low mg/L quantities in engineered strains [27–31]. This may be due to limitations in pools of metabolite intermediates, such as geranylgeranyl diphosphate (GPP), or the inherent toxicity and volatility of the monoterpenes themselves [32]. However, progress has been made in *E. coli*, exemplified by the production of limonene at 605 mg/L in extensively engineered strains harboring an optimized heterologous mevalonate terpene biosynthetic pathway [32–35]. Other monoterpenes including pinene, myrcene, geraniol and sabinene have also been engineered in *E. coli* [36–39]. *S. cerevisiae* has also been used to produce monoterpenes, but their production remains challenging due to the deficiency in the production of the key monoterpene intermediate GPP. A dual GPP/FPP synthase, encoded by the *S. cerevisiae ERG* 20 gene, produces 15 carbon FPP (farnesyl diphosphate) directly from dimethylallyl pyrophosphate (DMAPP) and isoprenyl diphosphate (IPP) without releasing the upstream intermediate 10 carbon GPP, which results in very low levels of GPP available for monoterpene production [40, 41]. Strategies have been developed to work around this issue, such as the replacement of *ERG20* with a mutant FPP synthase that has greater GPP activity; but these metabolic bottlenecks highlight the challenges of working with a host organism that does not naturally make high levels of terpenes [31, 40–43].

In addition to the metabolic hurdles that must be overcome for using model organisms like *S. cerevisiae* to make terpenes, bioconversion of lignocellulosic biomass also poses challenges for *S. cerevisiae* due to the fact that it is composed primarily of cellulose and hemicellulose which are polysaccharides of glucose, xylose and other sugars [44–46]. However, *S. cerevisiae* does not naturally utilize xylose and therefore requires additional engineering and rounds of optimization to enable its efficient utilization, which further complicates the efforts to produce desired bioproducts. Although xylose utilization pathways had been engineered into *S. cerevisiae* over two decades ago, optimization of efficient xylose utilization in the presence of glucose is still not satisfactory [46]. Furthermore, lignocellulose deconstruction using common pretreatment and saccharification approaches can produce inhibitory byproducts such as furfural, 5-hydroxymethylfurfural and phenols, which could reduce growth and productivity of the model yeast.

One strategy to overcome some of these barriers would be to choose a production host that naturally consumes lignocellulosic sugars, has tolerance to inhibitory compounds, and has high flux through key metabolic pathways used to produce the desired bioproducts [47–49]. Here, we explore the feasibility of using the carotenogenic red yeast *Rhodosporidium toruloides* IFO0880 to produce monoterpenes from lignocellulose. *R. toruloides* has several unique properties that make it a more appealing host for biofuel and bioproduct production than the model organisms *E. coli* and *S. cerevisiae*. *R. toruloides* can simultaneously utilize a broad range of carbon sources, including glucose and xylose derived from lignocellulose. In addition, it also has excellent tolerance towards inhibitory compounds found in lignocellulosic hydrolysates [49]. Moreover, *R. toruloides* is red pigmented due to the accumulation of high levels of carotenoids [50–52], which indicates that it has an efficient metabolic flux through the mevalonate pathway and therefore may be more amenable to producing non-native terpenes. Additionally, transgenic *R. toruloides* strains are notably stable due to the random integration of heterologous genes into its genome at high copy number using non-homologous end-joining (NHEJ) [53–56]. Overall, these characteristics suggest that *R. toruloides* may be an ideal host for the conversion of lignocellulose into terpene-based bioproducts.

**Fig. 1:**
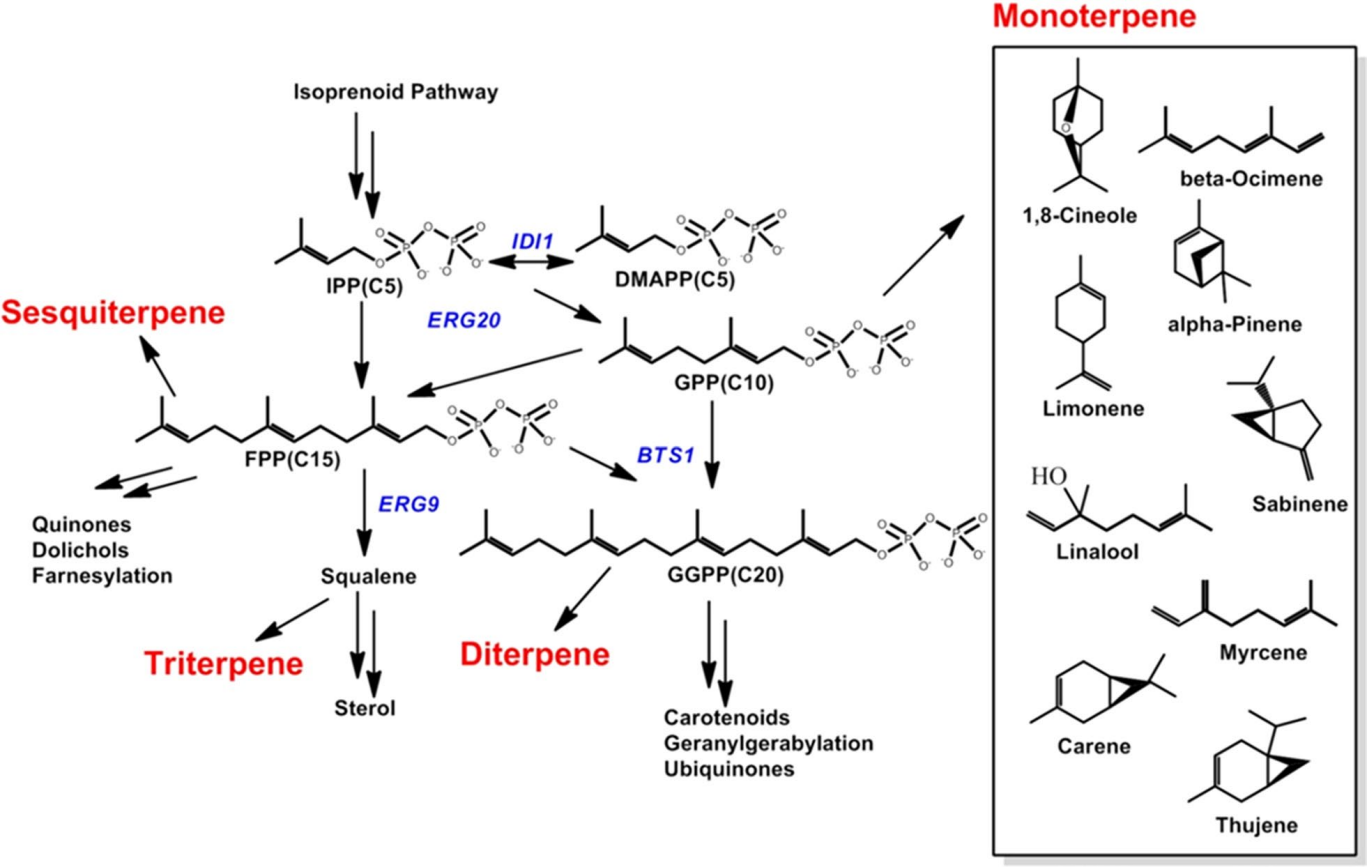
Schematic diagram of *R. toruloides* native mevalonate MVA pathways. The native MVA pathway provides the opportunity for diverting the intermediate GPP from the MVA pathway toward the biosynthesis of heterologous monoterpene bioproducts

There are two known pathways for terpene biosynthesis, the cytosolic mevalonate (MVA) pathway and the methylerythritol-phosphate pathway (MEP) pathway [57]. Typically, fungi and animals harbor the MVA pathway, while the MEP pathway predominates in bacteria [34]. Like most fungi, *R. toruloides* utilizes the MVA pathway to produce sterols and C40 carotenoids. The fact that it makes carotenoids indicates that *R. toruloides* produces all of the metabolic intermediates needed to make shorter chain terpenes, such as C5 hemiterpenes, C10 monoterpenes, C15 sesquiterpenes, etc. and provides an opportunity to make simply ‘drop-in’ expression constructs harboring a terpene synthase for a specific terpene biofuel or bioproducts. This study focuses specifically on monoterpenes, as illustrated in Fig. 1.

## Results

**Fig. 2:**
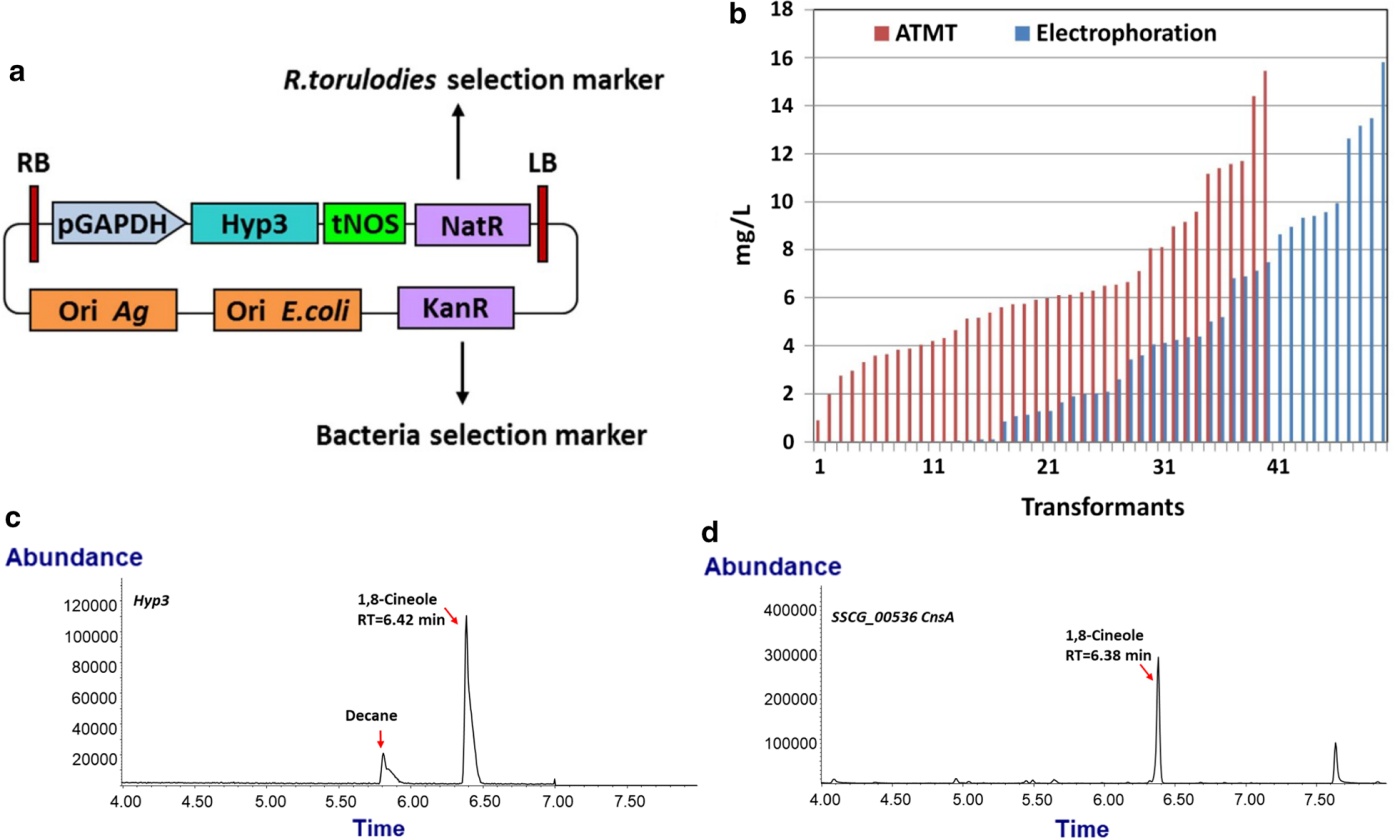
Cineole production in *R. toruloides*. **a** Binary vector containing the cineole synthase designed for genome integration in *R. toruloides* through the ATMT transformation method, which could also be used as template for PCR amplification to transform *R. toruloides* through the electroporation transformation method. **b** The comparison of cineole production in *R. toruloides* strains generated by the two transformation methods. **c** GC chromatographs of *R. toruloides* engineered for expression of the cineole synthase *Hyp3* using a dodecane overlay, and **d** *SSCG_00536 CnsA S*PME sample

The monoterpene synthases (MTS) selected for this study are listed in Table 1. Codon optimized MTS genes were cloned into a binary vector designed for *Agrobacterium tumefaciens* mediated transformation (ATMT) where expression is driven by the glyceraldehyde-3-phosphate dehydrogenase promoter (*pGAPDH*). An example of the vector harboring the *Hypoxylon* sp. *E74060B Hyp3* 1,8-cineole MTS gene is shown in Fig. 2a. These constructs can be used to randomly integrate transgenes into the genome of *R. toruloides* IFO0880 by ATMT or electroporation depending on how they are prepared, which adds flexibility to the engineering strategies. Sixteen constructs harboring MTS genes for the production of nine different monoterpenes were transformed into *R. toruloides* by either ATMT or electroporation and at least twenty individual transformants from either transformation approach were analyzed for production of target monoterpenes by GC–MS. The relative efficiency of these two transformation methods was compared using the 1,8-cineole MTS and is described in detail below.

**Table 1:** Summary of monoterpene synthase that have been tested for monoterpene production in *R. toruloides*

Product	Gene name	Organism	Gene bank Access number	Enzyme kinetic for GPP	Product in *R. toruloides*		References
					Dodecane overlay	SPME	
1,8-Cineole	*Hyp3*	*Hypoxylon* sp. *E74060B*	KJ433271.1	K_m_ = 2.5 ± 0.6 µM K_cat_ = 0.295 S^−1^	+++	+++	[58]
	*SSCG_00536* *CnsA*	*Streptomyces clavuligerus*	DS570626.1	K_m_ = 0.17 µM K_cat_ = 0.079 S^−1^	++	++	[59]
Ocimene	*ama0e23*	*Antirrhinum majus*	AY195607.1	NA	ND	++	[60]
	*LcTPS1*	*Licea cubeba*	HQ651178.1	NA	ND	ND	[75]
Limonene	*ag10*	*Abies grandis*	AF006193.1	NA	ND	+	[61]
Pinene	*PT30*	*Pinus taeda*	AF543530.1	K_m_ = 47 ± 9 µM	ND	++	[62]
	*AG3.18*	*Abies grandis*	U87909.1	K_m_ = 6 µM	ND	+	[61, 76]
Myrcene	*amaOc15*	*Antirrhinum majus*	AY195608.1	NA	ND	ND	[60]
	*ama1e20*	*Antirrhinum majus*	AY195609.1	NA	ND	ND	[60]
	*AG2.2*	*Abies grandis*	U87908.1	NA	ND	ND	[77]
Linalool	*PaTPS*-*Lin*	*Picea Abies*	AY473623.1	NA	ND	ND	[78]
Sabinene	*RlemTPS2*	*Citrus jambhiri*	AB266585.1	NA	ND	ND	[79]
	*SabS1*	*Salvia pomifera*	DQ785794.1	K_m_ = 7.4 µM	++	++	[63, 64]
Carene	*PaJF67*	*Picea abies*	AF461460	NA	ND	ND	[80]
	*TpsB*	*Salvia stenophylla*	AF527416.1	NA	ND	++	[65]
Thujene	*LcTPS2*	*Licea cubeba*	HQ651179.1	NA	ND	ND	[75]

ND, not detectable; +, detectable < 1 mg/L; ++, < 5 mg/L; +++, 5–20 mg/L

Two different analytical approaches were used to maximize the chances of detecting monoterpene product: (i) the use of an organic dodecane overlay to extract monoterpenes from liquid cultures and (ii) Solid Phase Micro Extraction (SPME) of monoterpenes from the headspaces of solid medium cultures (Table 1). The first approach has a monoterpene detection sensitivity of greater than 1 mg/L, while the SPME analysis is more sensitive and can detect trace levels of monoterpenes. The production and analysis of each monoterpene is described in detail in the following sections.

### Comparison of electroporation and ATMT transformation methods using the 1,8-cineole MTS

Two types of transformation methods have been established in *R. toruloides*, electroporation and ATMT, each using a fundamentally different method for introducing recombinant DNA into the cell. The *Hypoxylon* fungal 1,8-cineole *Hyp3* MTS gene (described in more detail in the next section) was transformed using both methods in order to select the most reliable method for subsequent transformations. Since both transformation methods established in *R. toruloides* randomly integrate variable copies of heterologous DNA into the genome via NHEJ, a number of transformants must be screened in order to identify the integrant with the optimal copy number and integration site to produce the maximum detectable amount of the target monoterpene. To compare the two transformation methods, 1,8-cineole titers were determined for 40 and 50 independent transformants from ATMT and electroporation, respectively. It was found that all of the ATMT transformants and 70% of electroporation transformants produced 1,8-cineole in dodecane overlaid liquid cultures (Fig. 2b). Despite the difference in the number of productive transformants, both methods generated transformants that produced the same maximum titer of 1,8-cineole (see below). These results suggest that fewer transformants need to be screened using the ATMT method to identify highly productive strains, so ATMT method was employed to transform the remaining MTS constructs.

### Monoterpene production in *R. toruloides*

The *Hypoxylon* fungal 1,8-cineole synthase *Hyp3* is the first identified fungal monoterpene synthase that has the conserved critical asparagine residues commonly found in plant synthase [58]. Overexpression of *HYP3* in *R. toruloides* yielded many transformants that produced 1,8-cineole in dodecane overlay cultures. The highest cineole titer obtained by electroporation was 15.45 mg/L, and 15.81 mg/L by ATMT (Fig. 2b). A single major product was detected at a retention time (RT) of 6.42 min with a MS spectrum that is identical to an authentic 1,8-cineol standard, indicating that 1,8-cineole is the sole product of this MTS (Fig. 2c). Shaw et al. illustrated that *Hyp3* produced minor amounts of d-limonene (about 5% of the total product) in *E. coli*, but only 1,8-cineole was detected in *R. toruloides*.

A second 1,8-cineole synthase encoded by the *Streptomyces clavuligerus* gene *SSCG_00536 CnsA* was also screened. *CnsA* is the first identified bacterial monoterpene cyclase that catalyzes the direct conversion of GPP into 1,8-cineole [59]. When the *CnsA* gene was expressed in *R. toruloides*, only trace amounts of 1,8-cineole were detected in the dodecane overlay of a liquid culture. By SPME analysis, a major 1,8-cineole product with RT of 6.38 min was detected (Fig. 2d).

**Fig. 3:**
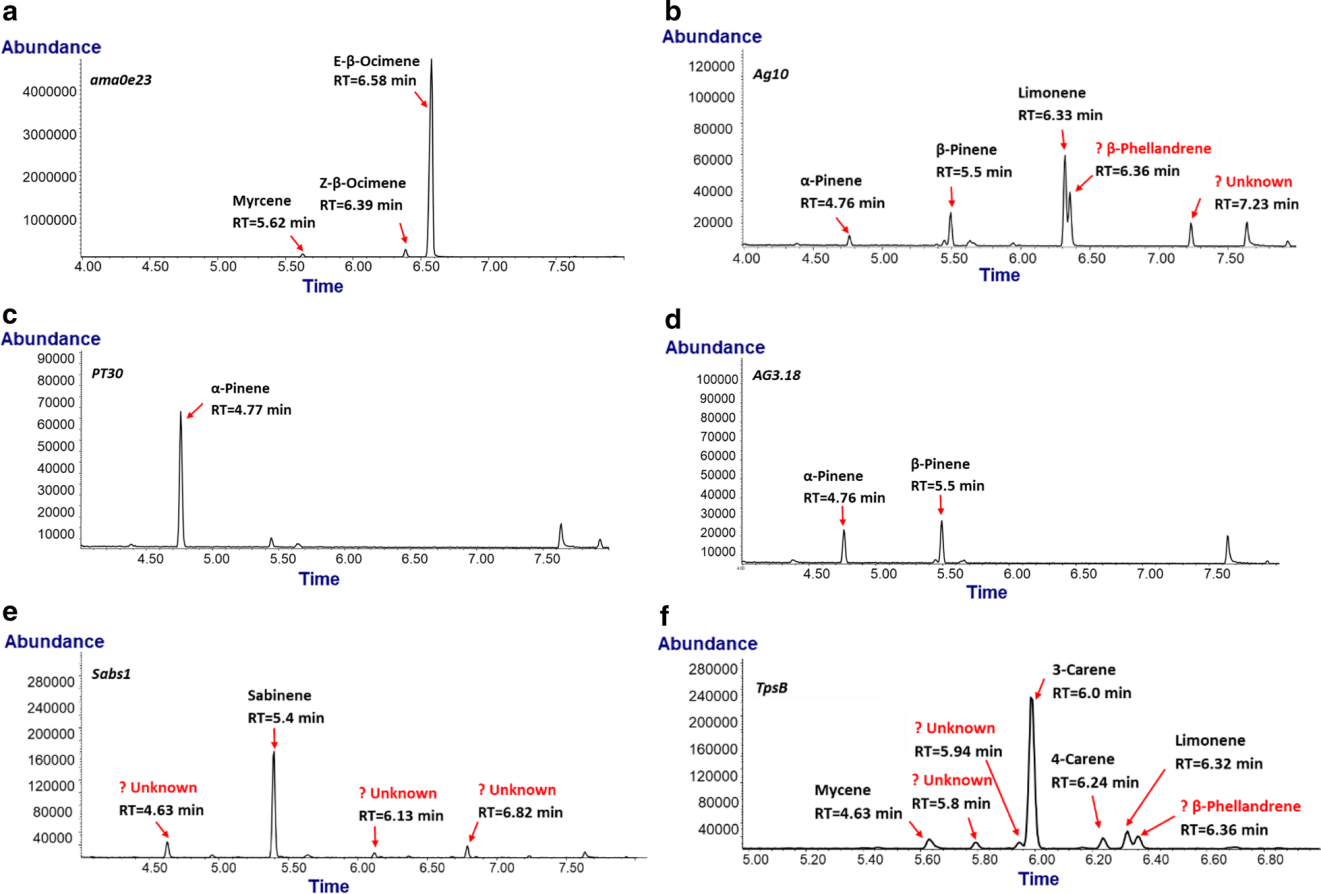
GC chromatographs of *S*PME samples from engineered *R. toruloides*. Expression of **a** the Ocimene synthase *ama0e23*, **b** limonene synthase *Ag10*, **c** pinene synthase *PT30*, **d** *Ag3.18*, **e** sabinene synthase *SabS1* and **f** carene synthase *TpsB*. Compounds that could be identified with authentic standards are labeled above the peaks of GC chromatograph. Peaks that might be from a monoterpene based on mass spectrum patterns, but lacking authentic standards, are labeled as unknown compounds

A truncated Snapdragon *Antirrhinum majus* (E)-β-Ocimene Synthase (*ama0a23*) was expressed in *R. toruloides*, producing (E)-β-ocimene as a major product, and (Z)-β-ocimene and myrcene as minor products, detected by SPME (Fig. 3a). These results are consistent with a study in which the cell free extracted MTS protein produced by *E. coli* was incubated with GPP and formed (E)-β-ocimene (97%), (Z)-β-ocimene (2%), and myrcene (1%) [60]. The *Licea cubeba* ocimene synthase *LcTPS* was also overexpressed in *R. toruloides*, but no product could be detected by either dodecane overlay or SPME analysis.

The grand fir (*Abies grandis*) (4S)-Limonene synthase *Ag10* was overexpressed in *R. toruloides* and SPME analysis detected a major peak of limonene and minor peaks of α-pinene and β-pinene (Fig. 2b). Two additional compounds with mass fragmentation patterns indicative of monoterpenes were detected, but their identities could not be clearly determined due to a lack of authentic standards. Bohlmann et al. functionally characterized *Ag10* and also detected limonene, α-pinene, and β-pinene, as well as β-phellandrene [61]. The mass spectrum of one of the unidentified products in *R. toruloides* was examined with the Wiley MS database library probability-based matching algorithm, and the results suggest that it is likely β-phellandrene. No match could be found for the second unidentified compound found.

Two pinene synthases, *Pinus taeda PT30* and *Abies grandis AG3.18,* were expressed in *R. toruloides* and examined by SPME as shown in Fig. 3c and d, respectively. A single compound, α-pinene, was produced by *R. toruloides* transformants expressing *PT30* gene, while both α-pinene and β-pinene were produced by transformants expressing the *AG3.18* gene. These results are consistent with previous characterizations of cell-free extracts of these enzymes expressed in *E. coli* supplied with GPP as substrate [61, 62].

Two sabinene synthases, *Citrus jambhiri RlemTPS2* and *Salvia pomifera Sabs1*, were expressed in *R. toruloides*. No detectable sabinene was produced by strains expressing *RlemTPS2*, while SPME analysis of the *Sabs1* strains detected sabinene and several other minor products (Fig. 3e). The three minor peaks could not be identified but produced mass fragmentation patterns indicative of monoterpenes (Fig. 3e). In a previous study of the *Sabs1* recombinant protein using GPP as substrate, several olefin monoterpenes were identified, including sabinene (63%), γ-terpinene (21%), terpinolene (7.0%), and limonene (6.5%) [63]. In contrast, Kampranis et al. reported that *Sabs1* produced sabinene and myrcene [64], indicating that this enzyme can produce different products depending on the experimental conditions, making it difficult to speculate on the identity of other monoterpenes produced in *R. toruloides* along with sabinene.

Two carene synthases, *Picea abies PaJF67* and *Salvia stenophylla TpsB*, were expressed in *R. toruloides* and only transformants expressing *TpsB* produced monoterpenes (Fig. 3f). A major peak of 3-carene was detected and verified with an authentic standard, while minor amounts of mycene, 4-carene, and limonene were also produced. Three additional compounds with monoterpene mass fragmentation patterns were also detected but could not be identified. And examination of the mass spectrum using the Wiley MS database library probability-based matching algorithm suggests that one of these unidentified peaks may be β-phellandrene, which agrees with a previous study of characterization of this enzyme. In that study, cell free enzyme assay of recombinant *TpsB* expressed in *E. coli* revealed that this enzyme can convert GPP to (+)-3-carene (73%), (−)-limonene (13%), myrcene (6%), 4-carene (4%), and β-phellandrene (1%) [65].

### Measuring the GPP and FPP pools in *R. toruloides* by over-expression of the FaNES1 gene

**Fig. 4:**
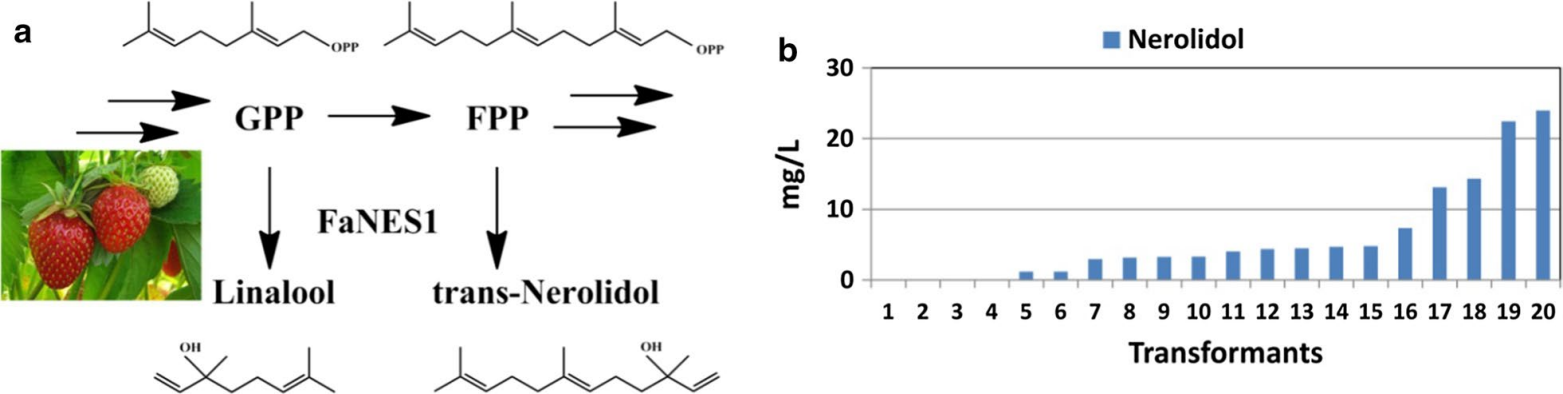
The strawberry-derived bifunctional enzyme FaNES1 was expressed in *R. toruloides* to examine the in vivo GPP and FPP metabolite pools. **a** FaNES1 can covert GPP into linalool and FPP into nerolidol. **b** Only nerolidol was produced in *R. toruloides*, with no detectable linalool

The expression of 16 MTS genes that are able to produce 9 different major monoterpenes in *R. toruloides* revealed that most of the MTS enzymes did not produce significant amounts of product. Only the *Hypoxylon Hyp3* enzyme could drive the production of appreciable amounts of 1,8-cineole, and even then, only in mg/L quantities. These results suggest that, like *S. cerevisiae*, the GPP pool in *R. toruloides* may have been limiting monoterpene production. In *S. cerevisiae* GPP is converted directly to FPP by *ERG20*. To assess whether this phenomenon also occurs in *R. toruloides*, the ratio of these two metabolites was determined. A low GPP/FPP ratio would indicate that GPP is limiting. A bifunctional linalool (monoterpene/GPP) and nerolidol (sesquiterpene/FPP) synthase *FaNES1* from strawberry was expressed and the ratio of these GPP/FPP-derived products was determined. A total of 20 independent transformants were examined, and while nerolidol was detected in several of them (up to 24 mg/L), no linalool was detected (Fig. 4). This observation is consistent with what was found in *S. cerevisiae* and suggests that the low level of GPP in *R. toruloides* is limiting monoterpene production and further engineering of the MEV pathway would be required to elevate monoterpene titers beyond what was observed in this study [40].

### Conversion of lignocellulose hydrolysate to 1,8-cineole

**Fig. 5:**
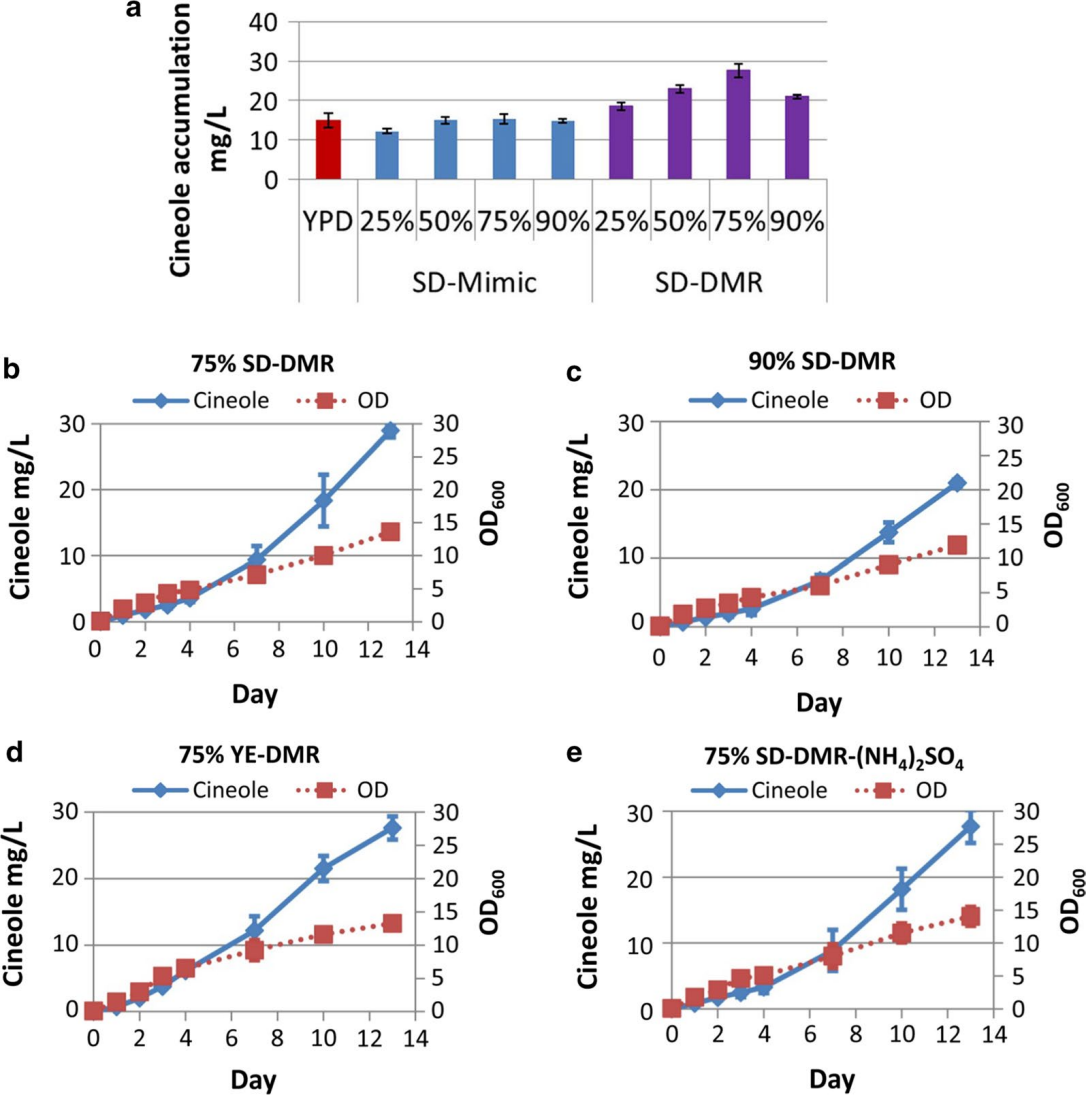
1,8-cineole production from DMR-EH hydrolysate in shake flasks. Cineole was produced in *R. toruloides* strain 17-3 in DMR-EH hydrolysate or a mock hydrolysate medium containing the same glucose and xylose concentrations (**a**). Cells grown in YPD medium served as the control (**a**). Comparison of cell growth and cineole production over 13 days in 75% DMR-EH with SD as the nitrogen source (**b**), 90% DMR-EH with SD as the nitrogen source (**c**), 75% DMR with yeast extract as the nitrogen source, and **d** 75% DMR-EH with SD and ammonium sulfate as the nitrogen source (**e**)

**Fig. 6:**
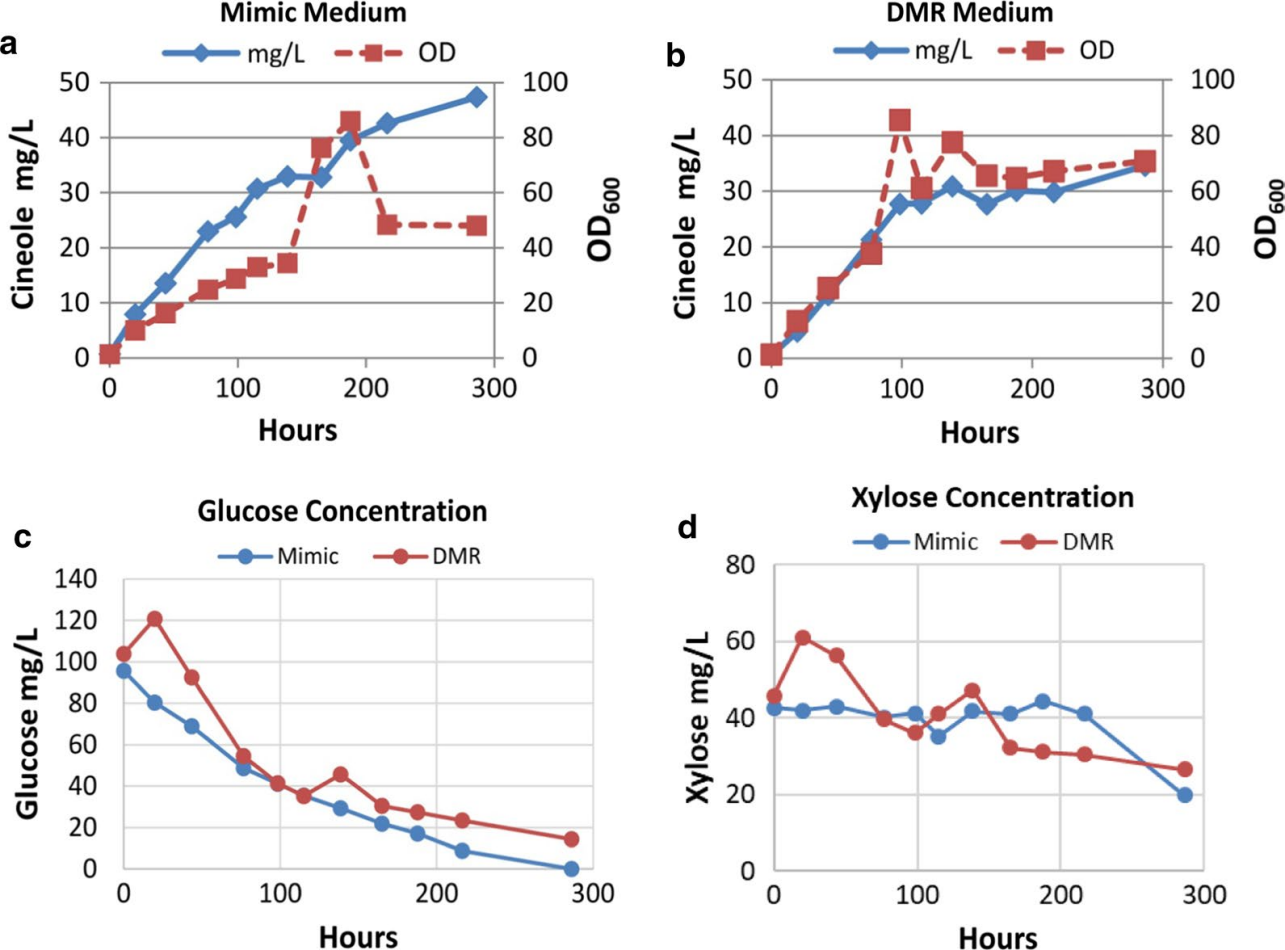
1,8-cineole production in DMR-EH hydrolysate in a 2L bioreactor. *R. toruloides* was cultivated in **b** 75% DMR-EH hydrolysate or **a** mock medium with the same amounts of glucose and xylose, each supplemented with 10 g/L yeast extract as a nitrogen source

To evaluate the ability of *R. toruloides* to convert lignocellulose into monoterpenes, the strain producing the highest levels of 1,8-cineole in rich media (15.81 mg/L) was cultivated in the corn stover DMR-EH (Deacetylated, Mechanically Refined, Enzymatically Hydrolyzed) hydrolysate supplemented with synthetic defined medium (SD). The hydrolysate was diluted with water to different percent volumes to determine the optimal hydrolysate concentration for 1.8-cineole production. The highest 1,8-cineole titer of 27.64 mg/L was achieved in 75% (v/v) DMR-EH hydrolysate (Fig. 5a, b). Overall, the production of 1,8-cineole in hydrolysate medium was higher than in mock medium containing equivalent amounts of sugars or in YPD medium. The 1,8-cineole production titer in 90% (v/v) DMR (21 mg/L) was found to be lower than in 75% (v/v) DMR (Fig. 5a–c), indicating possible mild growth inhibition or nutrient limitation. *R. toruloides* grew slower in 90% hydrolysate than in the 75% (v/v) hydrolysate, with a final OD_600_ of 11.9 and 13.3, respectively (Fig. 5b, c). 1,8-cineole production was also tested in 75% (v/v) DMR hydrolysate supplied with different nitrogen sources, using either 10 g/L yeast extract (YE) or SD with additional 2 g/L ammonium sulfate. Although *R. toruloides* grew faster initially in both media compared with the original hydrolysate, it eventually reached a similar OD_600_ in each medium (Fig. 5b–e). Finally, production of 1,8-cineole from lignocellulose was demonstrated in a 2L batch fermenter with 75% (v/v) DMR-EH hydrolysate or a mock hydrolysate containing 10 g/L YE. Both glucose and xylose consumption was observed in the cultivation, and 1,8-cineole titers reached 34.6 mg/L in DMR-EH hydrolysate and 47.3 mg/L in mock hydrolysate (Fig. 6).

### Fuel properties of 1,8-cineole

Previous research suggests that 1,8-cineole is potentially a good CI and SI fuel [12–16]. To explore the SI fuel properties of this compound in more detail, two important metrics relevant to SI fuels, research octane number (RON) and motor octane number (MON) were measured. Comparisons of these and other relevant fuel properties between 1,8-cineole and the well-known SI fuel additive ethanol, are shown in Table 2. While ethanol has a higher RON (109) and Sensitivity (19) relative to 1,8-cineole (99, 8), its high oxygen content, low energy density, and hygroscopic nature are less ideal for drop-in fuels. The fact that 1,8-cineole has low water solubility (3.5 g/L), high energy density (33.5 MJ/L), and RON/MON/Sensitivity values within the range of modern gasolines suggests that it is a good potential drop-in SI fuel. With an AKI defined as (RON + MON)/2 of ~ 95, pure 1,8-cineole would qualify as a “premium” fuel under the current gasoline standards.

**Table 2:** Comparisons of the relevant fuel properties of the 1,8-cineole (eucalyptol) with ethanol

	1,8-Cineole	Ethanol
Research octane number (RON)	99.2^a^	109^a^
Motor octane number (MON)	91.0^a^	90^a^
Sensitivity (RON-MON)	8.2	19
Energy density [MJ/L]	33.5^c^	20.2^c^
Heat of vaporization [kJ/kg]	255^c^	919^c^
Vapor pressure [kPa @25 °C]	0.25^c^	7.833^c^
Water solubility [g/L @21 °C]	3.5^c^	Fully miscible^c^
Oxygen content [% of total mass]	10.4	34.7^c^
Anti-knock index AKI = ½(RON + MON)	95.1	99.5^c^
Boiling point [°C]	176^c^	78.5^c^
Freez point/melting point [°C]	1^c^	− 114^c^
Flash point [°C]	49^c^	14^c^
OSHA hazards category	2^c^	2^c^

^a^Octane numbers for RON and MON determined by ASTM D2699 and D2700 respectively in collaboration with Intertek Group plc in Benicia CA

^b^Energy density based on the lower heating values (LHV) first reported by Wallner et al. [81] and later in the NREL fuel properties database [74]

^c^Physical properties gathered from the NREL Co-optima fuel properties database [74]

## Discussion

In this study, we investigated *R. toruloides* as a potential production host for terpene-based biofuels. Sixteen monoterpene synthases producing nine different target monoterpenes were evaluated and a total of seven monoterpenes were successfully produced in *R. toruloides*. The highest monoterpene titer achieved was 48 mg/L of 1,8-cineole; while this is impressive for a wild-type organism, the natural flux through the essential GPP monoterpene precursor metabolite may still be limiting. The fact that the bifunctional mono/sesquiterpene synthase used in this study to assess the GPP/FPP ratio produced only the FPP-derived sesquiterpene nerolidol, suggested that *R. toruloides*, like other yeast, produces low amounts of the monoterpene precursor GPP [40]. Examination of the genome indicated that *R. toruloides* does not have a dedicated GPP synthase, but like *S. cerevisiae*, it has a single GPP/FPP synthase (*ERG20*) that also appears to preferentially produce FPP at the expense of GPP. In addition, this organism has a GGPP synthase, which may also potentially use GPP as a substrate, as has been demonstrated for other GGPP synthases [66]. Therefore, between these two enzymes, it is not surprising that GPP pool is low. One strategy to enhance GPP levels would be to knock-out the GGPP synthase and replace the native *ERG20* with a mutated version that preferentially produces GPP, as demonstrated in other organisms.

Beyond balancing the MVA pathway flux to favor monoterpene production, another target to optimize are the terpene enzymes. The *Hypoxylon* sp. *Hyp3* gene used to produce the highest titer of 1,8-cineole is a fungal gene and has good enzyme kinetic parameters with K_m_ of 2.5 ± 0.6 µM and K_cat_ of 0.295 S^−1^ [58]. This enzyme is derived from fungi, which means that it does not have a N-terminal plastid signal sequence like plant monoterpene or diterpene synthases, which are often expressed in chloroplast and need to be truncated to be in an active form. Many of the other terpene synthases tested in this study were from plants and were truncated, but it is often difficult to truncate the protein at the right position. For example, a previous study examined a series of N-terminal truncations to produce a “pseudomature” form of limonene synthase lacking the plastid signal sequence, and found that a N-terminal arginine pair was important for the function of the synthase as it plays a role in substrate binding and ionization [67]. Therefore, it is possible that the truncation sites chosen for some of the monoterpene synthases in this study were suboptimal, and that alternative truncations would result in better performance. These alternative truncations will be pursued in future studies along with detailed kinetic measurements of the enzymes to gain a better understanding of the impact of signal peptides on enzyme activities.

*Rhodosporidium toruloides* is an oleaginous yeast that can accumulate triacylglycerides (TAGs) up to 70% of its weight when starved for nitrogen [68, 69]. Strategies that limit driving carbon from the central metabolite acetyl-CoA towards lipid biosynthesis, such as deletions of TAG biosynthetic genes, could potentially allow for more carbon to be naturally diverted into the MVA pathway, resulting in higher terpene production. This diversion of flux could be further enhanced by overexpressing MVA pathway genes, especially those upstream of the GPP intermediates (*ERG10*, *ERG13*, *ERG12*, *ERG8*, *HMGR*, *IDI*), or transcription factors that positively regulate the pathway. The natural production of carotenoids in this organism can potentially be used as a tool to detect enhanced flux through the MVA pathway by screening for colonies with increased red pigmentation. This tool could be used for both the aforementioned targeted engineering strategies in addition to traditional mutagenesis screening. Process optimization could also be used to divert carbon away from lipid biosynthesis toward the MVA pathway, such as optimizing the carbon/nitrogen ratio, examining different carbon sources, nutrients, temperatures, pHs, osmolite concentrations, etc. Taken together, these strategies will likely improve monoterpene production significantly, and will be the focus of future studies.

In addition to the natural ability to produce carotenoids, the other major attractive characteristics of *R. toruloides* is its ability to consume a wide range of carbon sources, including the major components derived from lignocellulose (glucose and xylose), suggesting that lignocellulosic hydrolysates may be an ideal carbon source for this organism. To explore this concept further, *R. toruloides* was grown on a DMR-EH corn stover hydrolysate containing glucose, xylose and acetate and was shown to grow well and produce higher titers of 1,8-cineole than from a mock hydrolysate or rich medium. DMR-EH hydrolysate is produced in a process where lignocellulosic biomass (corn stover) was deacetylate in dilute alkali, mechanically refined, and enzymatically hydrolyzed to produce high concentrations of monomeric sugars [70]. According to the 2016 D.O.E. Billion-Ton Report, there are 1.2 to 1.5 billion tons of dry lignocellulosic biomass available in the US, which, if converted into biofuel, could be used to displace 30% of current domestic petroleum consumption [5]. Our results suggest that *R. toruloides* can be a good addition to the lignocellulosic biofuel portfolio.

Of the monoterpenes tested, 1,8-cineole was produced at the highest titers in *R. toruloides*. Therefore, we examined its fuel properties in more detail and found that it may be a good SI fuel, especially for downsized, boosted SI engines. The RON/MON/Sensitivity of 1,8-cineol are within the range of premium gasoline and its other properties, like high energy density, make it an appealing biofuel candidate. 1,8-cineole has an energy density that is 5–8% higher than gasoline, while ethanol has an energy density that is 34–36% lower than gasoline. This difference would allow a vehicle to get nearly twice the mileage per gallon (MPG) on a tank of 1,8-cineole as it would on a tank of ethanol assuming no change in MPG due to the lower octane and heat of vaporization (HoV) of 1,8-cineole. While the slightly lower octane and HoV will lower the potential engine efficiency of 1,8-cineole compared to ethanol, the much higher energy density would compensate for it. The only major downside of 1,8-cineole as a fuel is its high freezing point of 1 °C, limiting its use as a drop-in fuel to warm climates. However, this property could be modified by blending 1,8-cineol into gasoline itself, or with the addition of another biofuel molecule that can decrease its freezing point. Ethanol may in fact be a good candidate, and the blend could be formulated to maximize each component’s favorable fuel properties. Along those lines, previous reports have shown that when used as an additive in ethanol-gasoline SI fuel blends, 1,8-cineole can reduce fuel volatility, prevent phase separation, and improve RON [12–14]. Also, 1,8-cineole has been blended into diesel up to 15% by volume for use in a four stroke single cylinder diesel compression ignition (CI) engine, and showed promising fuel emission characteristics [15]. Taken further, a recent study modified a single cylinder diesel CI engine to utilize 100% eucalyptus oil (primarily composed of 1,8-cineole) as fuel [16]. Therefore, 1,8-cineole appears to be a dual-purpose molecule that can be potentially used for multiple different engine types, including the two most common engines (SI and CI) found in vehicles in the US.

## Conclusions

In this study, we have successfully demonstrated the use of *R. toruloides* as a production host for the conversion of lignocellulosic biomass into monoterpenes, many of which have potential applications as biofuels. The investigation of one particular biofuel molecule, 1,8-cineole, revealed that it may be a promising SI and CI fuel.

## Methods

### Chemical reagents and oligonucleotide

Phusion DNA polymerase and T4 Ligase were purchased from New England Biolabs (Ipswich, MA, USA). The Fast Digest restriction enzymes were purchased from Thermo Fisher Scientific (Waltham, MA, USA). The DNA oligonucleotides were synthesized by Elim Biopharm (Hayward, CA, USA). PCR amplification with Phusion High-Fidelity DNA Polymerase (Thermo Fisher Scientific, Waltham, MA, USA) was performed according to the manufacturer’s instructions. *Escherichia coli* DH5α competent cells for chemical transformation were prepared with Mix and go *E. coli* transformation buffer set (T3002, Zymo Research Irvine, CA, USA). Chemical transformation of *E. coli* was done according to the supplier’s instructions. The amplified PCR products were purified using DNA clean and concentrate kit. Plasmids were prepared using Zyppy Plasmid Miniprep Kit and yeast genomic DNA was extracted using the YeaStar Genomic DNA kit (Zymo research Irvine, CA, USA). Other chemical reagents used in this study were obtained from Sigma-Aldrich (St. Louis, MO, USA).

### Strains, plasmids and culture conditions

*Rhodosporidium toruloides* IFO0880 and *Agrobacterium* EHA 105 were obtained from Jeffrey Skerker at UC Berkeley [56]. *R. toruloides* was cultured in YPD medium at 30 °C, except for monoterpene production quantification where *R. toruloides* was cultured in YPD medium at 23 °C. Hemiterpene and monoterpene genes were designed to remove the chloroplast targeting peptides and were codon optimized for expression in *R. toruloides*. Codon optimized terpene synthases were synthesized by Genscript (Piscataway, NJ, USA), and then cloned into PGI2 binary vector [56], which were either used to transform to *R. toruloides* through agrobacterium mediated transformation, or serve as a template to PCR with primers P1: AGGGTTTTCCCAGTCACGACGTTG and P2: CATGATTACGAATTCGCCCTTTC amplify the terpene gene expression cassette along with the antibiotic selection maker cassette to transform into *R. toruloides* by electroporation. Detailed DNA sequence and vector map are in Additional file 1. YPD medium (yeast extract 10 g/L, peptone 20 g/L, and glucose 20 g/L) was used for routine growth of *R.* toruloides. *Agrobacterium tumefaciens* EHA 105 and *E. coli* strains were cultivated in LB broth (10 g/L tryptone, 5 g/L yeast extract, 10 g/L NaCl) at 30 °C and 37 °C, respectively. All solid media plates were prepared with 2% Bacto-Agar. For *A*. *tumefaciens* culture, the media is supplemented with 10 mg/L rifampicin to prevent contamination.

Pure DMR-EH corn stover hydrolysate containing 105 g/L glucose, 46 g/L xylose and 1.2 g/L acetic acid, was obtained from National Renewable Energy Laboratory (NREL). The hydrolysate was prepared through two rounds of centrifugation and filtration. The first centrifugation was performed at 14,000 rcf (at 4 °C for 35 min), and after the solids were separated, a second centrifugation was performed at 30,000 rcf (at 4 °C for 45 min). Afterwards, a PES filter cartridge was used for filtration, first at 0.45 μm^2^, then at 0.2 μm^2^. SD (synthetic defined) medium having 6.7 g/L yeast nitrogen base and 0.69 g/L CSM supplement was added to the hydrolysate as a nitrogen source. A mock hydrolysate was also formulated in SD with equivalent amounts of glucose and xylose.

Bioreactor cultivation was performed at Advanced Biofuels Process Demonstration Unit (ABPDU)as described previously [71]. Cineole production in *R. toruloides* was examined by batch fermentation in 2L bioreactors (BIOSTAT B, Sartorius, Germany) with extractive fermentation with 20% dodecane overlay. Fermentation parameters were set as 30 °C for temperature and 40% air saturation for dissolved oxygen. Dissolved oxygen was controlled by adjusting the agitation rate at a constant airflow. The pH of the culture started at 7.4 and was not adjusted and no substrate was added during fermentation. Foaming was controlled by adding 5% (v/v) Antifoam 204 as needed. The batch medium in the fermenter had the following components: 10 g/L yeast extract with 75% of DMR hydrolysate or xylose and glucose mixture equivalent to the 75% DMR-EH hydrolysate as the mock medium. Cell growth and cineole production was monitored by taking 5 mL samples at different time points during the cultivation.

### Transformation method

Two transformation methods, electroporation and agrobacterium-mediated fungal transformation were used to transform *R. toruloides*. Using PGI2-derived binary vectors [56] as template, a PCR amplification product having the terpene synthase and *R. toruloides* antibiotic selection cassette DNA were obtained using primers P1 and P2 (see Additional file 1: Table S1). After cleaning and purification, those DNA fragments could be used to transform directly to *R. toruloides* by electroporation. Electroporation was done in a 1 mm cuvette (1652083, Bio-Rad, Hercules, CA, USA) using a Gene Pulser Xcell Electroporation System (Bio-Rad). *R. toruloides* was subcultured into 50 mL medium from an overnight seed culture and grew until OD_600_ reached around 2 to 2.5. The culture was harvested and washed with ice cold 1 M sorbitol four times, and resuspended in 0.5 mL of ice cold 1 M sorbitol. Purified DNA (150 ng–500 ng) was electroporated using a Gene Pulser Xcell (Bio-Rad) with 1.5 kV electrical pulse, 200 ohms, 25 μF in 1 mm cuvette, and recovered in 1 mL YPD at 30 °C for 3 h and then plated onto YPD plates containing 100 mg/L Nourseothricin. Colonies formed after 3 days of incubation at 30 °C.

*Agrobacterium*-mediated fungal transformation protocol used in this study is based on a published method with some modifications [53, 54, 56, 72, 73]. pGI2-derived binary plasmids were first electroporated to *Agrobacterium* EHA105. *Agrobacterium* strain harboring the binary plasmid was cultured in 1 mL liquid LB medium supplemented with rifampicin (10 mg/L) and kanamycin (50 mg/L) and grew until OD_600_ reach to about 1 at 30 °C. The culture was pelleted and resuspended in 1 ml induction medium and shaked for approximately 24 h at 30 °C. The induction medium contained 100 μM acetosyringone, 3.9 g/L 2-(N-morpholino) ethane sulfonic acid, 1 g/L NH_4_Cl, 0.3 g/L MgSO_4_·7H_2_O, 0.15 g/L KCl, 0.01 g/L CaCl_2_, 0.75 mg/L FeSO_4_·7H_2_O, 0.144 g/L K_2_HPO_4_, 0.048 g/L NaH_2_PO_4_, 3.56 g/L glucose, and 0.01 g/L thiamine. *R. toruloides* was cultured in YPD medium until OD_600_ reached 1, and mixed with the induced *A. tumefaciens* cells in equal volume to a final volume of 1 mL. The mixture was spread on a Millipore membrane (HAWP 0.45 µm) and filtered by applying vacuum. The membrane was then placed onto an induction medium plate and co-cultured at 26 °C for 3 to 4 days. The cells on the membrane were then resuspended in YPD medium and plated onto YPD agar plate supplemented with 100 mg/L nourseothricin and 300 mg/L cefotaxime. Cefotaxime was used to kill the *A. tumefaciens* cells. Selection plates were incubated at 30 °C for 2 to 3 days until colonies formed.

### Terpene quantification

*Rhodosporidium toruloides* transformants were cultivated in 10 mL liquid YPD media, and after cells reached OD_600_ of about 1, 20% dodecane were added to the culture on the second day. Then, the dodecane phase was collected at different time points and diluted properly with ethyl acetate, and 1 µL of organic liquid was analyzed by Agilent GC–MS 6890 system with Agilent DB5 silica capillary column (30 m × 0.25 mm × 0.25 μm film thicknesses). For terpene identification by SPME (Solid Phase Microextraction) method, fibers were first conditioned according to the suppliers’ instruction. Then SPME fiber was exposed to the head-space of *R. toruloides* samples to extract volatile compounds and the absorbed samples were directly injected on GC–MS. The initial oven temperature was set at 60 °C for 2 min, ramped to 120 °C at 10 °C/min, and then ramped to 300 °C at 80 °C/min and held for 5 more minutes. Terpene quantification were calculated by comparison with the 1,8-cineole standard curve.

### Fuel property measurements

High purity 1,8 cineole samples were sent to Southwest Research Institute (SWRI) Fuels and Lubricants Research Division at San Antonio, Texas and were burned in certified CFR engines for research octane number (RON) and motor octane number (MON) according to ASTM D2599 and ASTM D2600a standards respectively. Reproducibility for the method is < 0.7 ON for both the RON and MON tests. All other properties for 1,8 cineole and ethanol have been previously reported in literature and were sourced from the Co-Optima Fuel Properties Database [74].

## Additional file

**Additional file 1.** DNA sequences of the monoterpene synthases.
