# Supplementary material for: Monoterpene production by the carotenogenic yeast Rhodosporidium toruloides
Source: Microb Cell Fact. 2019 Mar 18;18:54. doi: 10.1186/s12934-019-1099-8 (PMC6421710; doi:10.1186/s12934-019-1099-8)
Supplement: Supplementary file 1 — Additional file 1. DNA sequences of the monoterpene synthases. [file 12934_2019_1099_MOESM1_ESM.docx]

Monoterpene production by the carotenogenic yeast *Rhodosporidium toruloides*

Xun Zhuang^1^, Oliver Kilian^1^, Eric Monroe^1^, Masakazu Ito^3^, Mary Bao Tran-Gymfi^1^, Fang Liu^1^, Ryan W Davis^1^, Mona Mirsiaghi^2^, Eric Sundstrom^2^, Todd Pray^2^, Jeffrey M. Skerker^3,4^, Anthe George^1,5*^ and John M. Gladden^1,5*^

**Affiliations:**

1 Biomass Science and Conversion Technology, Sandia National Laboratories, 7011 East Ave, Livermore, CA 94551, USA.

2 Advanced Biofuels Process Development Unit (ABPDU), Lawrence Berkeley National Laboratory, 5885 Hollis St, Emeryville, CA 94608, USA Emeryville, CA

3 Energy Bioscience Institute, 2151 Berkeley Way, Berkeley, CA 94704, USA

4 Lawrence Berkeley National Laboratory, 1 Cyclotron Rd, Berkeley, CA 94720, USA

5 Deconstruction Division, Joint BioEnergy Institute / Sandia National Laboratories, 5885 Hollis St, Emeryville, CA 94608, USA Emeryville, CA

*Correspondence to: jmgladden@lbl.gov (J.M.G); [ageorge@lbl.gov](mailto:ageorge@lbl.gov) (A.G)

Email address for all Authors:

Xun Zhuang: zhuangxun@gmail.com

Oliver Kilian: oli.killian@gmail.com

Eric Monroe: emonroe@sandia.gov

Masakazu Ito: mskz@berkeley.edu

Mary Bao Tran-Gymfi: mbtrang@sandia.gov

Fang Liu: fanliu@sandia.gov

Ryan W Davis: [rwdavis@sandia.gov](mailto:rwdavis@sandia.gov)

Mona Mirsiaghi: mona.mirsiaghi@gmail.com

Eric Sundstrom: [esundstrom@lbl.gov](mailto:esundstrom@lbl.gov)

Todd Pray: tpray@lbl.gov

Jeffrey M. Skerker: skerker1@gmail.com

Anthe George: ageorge@lbl.gov

John M. Gladden: jmgladden@lbl.gov

DNA sequence of Monoterpene Synthase

Hyp3

ATGCGCCCCATCACGTGCTCGTTCGACCCGGTCGGCATCTCGTTCCAGACCGAGTCGAAGCAGGAGAACTTCGAGTTCCTCCGCGAGGCCATCTCGCGCTCGGTCCCCGGCCTGGAGAACTGCAACGTCTTCGACCCCCGCTCGCTCGGCGTCCCGTGGCCCACGTCGTTCCCTGCGGCGGCGCAGTCGAAGTACTGGAAGGACGCCGAGGAAGCGGCGGCGGAGCTGATGGACCAGATCGTCGCGGCGGCGCCTGGCGAGCAGGGCTCGCTCCCGGCGGAGCTGGCCGTCTCGGACAAGAAGGCGGCCAAGCGCCGAGAGCTGCTCGACACCTCCGTCTCGGCCCCCATGAACATGTTCCCTGCGGCGAACGCGCCTCGCGCGCGCATCATGGCCAAGGCGAACCTCCTCATCTTCATGCACGACGACGTCTGCGAGTACCAGTCGGTCCAGTCCACGATCATCGACTCGGCCCTCGCGGACACCTCGACGCCTAACGGCAAGGGCGCCGACATCCTCTGGCAGAACCGCATCTTCAAGGAGTTCTCGGAGGAGACGAACCGCGAGGACCCGGTCGTCGGCCCTCAGTTCCTCCAGGGCATCCTCAACTGGGTCGAGCACACGCGCAAGGCCCTCCCCGCGTCGATGACCTTCCGCTCGTTCAACGAGTACATCGACTACCGCATCGGCGACTTCGCCGTGGACTTCTGCGACGCCGCGATCCTCCTCACGTGCGAGATCTTCCTCACCCCCGCGGACATGGAGCCCCTCCGCAAGCTCCACCGCCTCTACATGACGCACTTCTCGCTCACCAACGACCTCTACTCGTTCAACAAGGAAGTCGTCGCCGAGCAGGAGACGGGCTCGGCCGTCATCAACGCGGTCCGCGTCCTGGAGCAGCTCGTGGACACCTCGACGCGCTCGGCCAAGGTCCTCCTCCGCGCGTTCCTCTGGGACCTCGAACTCCAGATCCACGACGAGCTGACGCGCCTCAAGGGCACGGACCTCACGCCTTCGCAGTGGCGCTTCGCTCGCGGCATGGTCGAGGTCTGCGCCGGCAACATCTTCTACTCGGCCACCTGCCTCCGCTACGCCAAGCCGGGCCTCCGCGGCATCTGA

MRPITCSFDPVGISFQTESKQENFEFLREAISRSVPGLENCNVFDPRSLGVPWPTSFPAAAQSKYWKDAEEAAAELMDQIVAAAPGEQGSLPAELAVSDKKAAKRRELLDTSVSAPMNMFPAANAPRARIMAKANLLIFMHDDVCEYQSVQSTIIDSALADTSTPNGKGADILWQNRIFKEFSEETNREDPVVGPQFLQGILNWVEHTRKALPASMTFRSFNEYIDYRIGDFAVDFCDAAILLTCEIFLTPADMEPLRKLHRLYMTHFSLTNDLYSFNKEVVAEQETGSAVINAVRVLEQLVDTSTRSAKVLLRAFLWDLELQIHDELTRLKGTDLTPSQWRFARGMVEVCAGNIFYSATCLRYAKPGLRGI*

*SSCG_00536*

ATGCCTGCGGGCCACGAGGAGTTCGACATCCCGTTCCCCTCGCGCGTCAACCCTTTCCACGCTCGCGCGGAGGACCGCCACGTCGCCTGGATGCGCGCGATGGGCCTCATCACGGGCGACGCGGCCGAGGCCACCTACCGCCGATGGTCGCCGGCCAAGGTCGGCGCTCGCTGGTTCTACCTCGCCCAGGGCGAGGACCTCGACCTCGGCTGCGACATCTTCGGCTGGTTCTTCGCCTACGACGACCACTTCGACGGCCCCACCGGCACCGACCCCCGCCAGACCGCGGCCTTCGTCAACCGCACCGTCGCCATGCTCGACCCGCGCGCGGACCCCACCGGCGAGCACCCCCTCAACATCGCCTTCCACGACCTCTGGCAGCGCGAGTCGGCCCCCATGTCGCCCCTCTGGCAGCGCCGAGCGGTGGACCACTGGACCCAGTACCTCACGGCCCACATCACGGAGGCCACCAACCGCACGCGCCACACCTCGCCTACCATCGCGGACTACCTCGAACTCCGCCACCGCACGGGCTTCATGCCGCCCCTCCTCGACCTCATCGAGCGCGTCTGGCGCGCGGAGATCCCCGCCCCCGTCTACACGACCCCGGAGGTCCAGACGCTCCTCCACACGACCAACCAGAACATCAACATCGTCAACGACGTCCTCTCGCTGGAGAAGGAAGAGGCGCACGGCGACCCGCACAACCTCGTCCTCGTCATCCAGCACGAGCGCCAGTCCACGCGCCAGCAGGCGCTCGCCACCGCCCGCCGAATGATCGACGAGTGGACCGACACCTTCATCCGCACGGAGCCCCGCCTCCCGGCCCTCTGCGGACGCCTCGGCATCCCCCTCGCCGACCGCACCTCGCTCTACACGGCGGTCGAGGGCATGCGCGCCGCCATCCGCGGCAACTACGACTGGTGCGCGGAGACGAACCGCTACGCCGTCCACCGCCCGACGGGCACCGGCCGCGCGACGACCCCCTGGTGA

MPAGHEEFDIPFPSRVNPFHARAEDRHVAWMRAMGLITGDAAEATYRRWSPAKVGARWFYLAQGEDLDLGCDIFGWFFAYDDHFDGPTGTDPRQTAAFVNRTVAMLDPRADPTGEHPLNIAFHDLWQRESAPMSPLWQRRAVDHWTQYLTAHITEATNRTRHTSPTIADYLELRHRTGFMPPLLDLIERVWRAEIPAPVYTTPEVQTLLHTTNQNINIVNDVLSLEKEEAHGDPHNLVLVIQHERQSTRQQALATARRMIDEWTDTFIRTEPRLPALCGRLGIPLADRTSLYTAVEGMRAAIRGNYDWCAETNRYAVHRPTGTGRATTPW*

*ama0e23*

ATGGGCTCGACCCCGCCCCCGTCGAAGCTCCACCAGGCGCTCTGCCTCAACGCCCACTCGACGTCGTGCATGGCGGAGCTGCCGATGGACTACGAGGGCAAGATCCAGGGCACCCGCCACCTCCTCCACCTCAAGGACGAGAACGACCCGATCGAGTCGCTCATCTTCGTGGACGCCACCCAGCGCCTCGGCGTCAACCACCACTTCCAGAAGGAGATCGAGGAGATCCTCCGCAAGTCGTACGCCACCATGAAGTCGCCCTCGATCTGCAAGTACCACACGCTCCACGACGTCTCGCTCTTCTTCTGCCTCATGCGCCAGCACGGCCGCTACGTCTCGGCCGACGTCTTCAACAACTTCAAGGGCGAGTCGGGCCGCTTCAAGGAAGAGCTGAAGCGCGACACCCGCGGCCTCGTCGAGCTGTACGAGGCCGCCCAGCTCTCGTTCGAGGGCGAGCGCATCCTCGACGAGGCCGAGAACTTCTCGCGCCAGATCCTCCACGGCAACCTCGCCTCGATGGAGGACAACCTCCGCCGATCGGTCGGCAACAAGCTCCGCTACCCGTTCCACAAGTCGATCGCGCGCTTCACGGGCATCAACTACGACGACGACCTCGGCGGCATGTACGAGTGGGGCAAGACGCTCCGCGAGCTGGCGCTCATGGACCTCCAGGTCGAGCGCTCGGTCTACCAAGAGGAGCTGCTCCAGGTCAGCAAGTGGTGGAACGAGCTGGGCCTCTACAAGAAGCTCACGCTCGCGCGCAACCGCCCTTTCGAGTTCTACATGTGGTCGATGGTCATCCTCACCGACTACATCAACCTCTCGGAGCAGCGCGTCGAGCTGACGAAGTCGGTCGCCTTCATCTACCTCATCGACGACATCTTCGACGTCTACGGCACCCTCGACGAGCTGATCATCTTCACGGAGGCGGTCAACAAGTGGGACTACTCGGCCACCGACACCCTCCCCGACAACATGAAGATGTGCTACATGACGCTCCTCGACACCATCAACGGCACGTCGCAGAAGATCTACGAGAAGTACGGCCACAACCCCATCGACTCGCTCAAGACGACCTGGAAGTCGCTCTGCTCGGCGTTCCTCGTCGAGGCCAAGTGGTCGGCCTCGGGCTCGCTCCCCTCGGCCAACGAGTACCTGGAGAACGAGAAGGTCAGCTCGGGCGTCTACGTCGTCCTCATCCACCTCTTCTTCCTCATGGGCCTCGGCGGCACCAACCGCGGCTCGATCGAGCTGAACGACACGCGCGAGCTGATGTCGTCGATCGCGATCATCGTCCGCATCTGGAACGACCTCGGCTGCGCCAAGAACGAGCACCAGAACGGCAAGGACGGCTCGTACCTCGACTGCTACAAGAAGGAGCACATCAACCTCACGGCGGCCCAGGTCCACGAGCACGCGCTCGAACTCGTCGCCATCGAGTGGAAGCGCCTCAACAAGGAGTCGTTCAACCTCAACCACGACTCCGTCTCGTCGTTCAAGCAGGCCGCCCTCAACTTCGCCCGCATGGTCCCCCTCATGTACTCGTACGACAACAACCGCCGAGGCCCCGTCCTGGAGGAGTACGTCAAGTTCATGCTCTCGGACTGA

MGSTPPPSKLHQALCLNAHSTSCMAELPMDYEGKIQGTRHLLHLKDENDPIESLIFVDATQRLGVNHHFQKEIEEILRKSYATMKSPSICKYHTLHDVSLFFCLMRQHGRYVSADVFNNFKGESGRFKEELKRDTRGLVELYEAAQLSFEGERILDEAENFSRQILHGNLASMEDNLRRSVGNKLRYPFHKSIARFTGINYDDDLGGMYEWGKTLRELALMDLQVERSVYQEELLQVSKWWNELGLYKKLTLARNRPFEFYMWSMVILTDYINLSEQRVELTKSVAFIYLIDDIFDVYGTLDELIIFTEAVNKWDYSATDTLPDNMKMCYMTLLDTINGTSQKIYEKYGHNPIDSLKTTWKSLCSAFLVEAKWSASGSLPSANEYLENEKVSSGVYVVLIHLFFLMGLGGTNRGSIELNDTRELMSSIAIIVRIWNDLGCAKNEHQNGKDGSYLDCYKKEHINLTAAQVHEHALELVAIEWKRLNKESFNLNHDSVSSFKQAALNFARMVPLMYSYDNNRRGPVLEEYVKFMLSD

*LcTPS1*

ATGCACAACGCGCTCCAGATCGCCCGCCGATCGGCCAACTACCACCCCTCGATCTGGGACCCCCAGTACATCGAGTCGCTCAAGTCGCCGTACGGCGACGAGTGCTTCGGCACGCGCCTGGAGAAGCTCAAGTTCGAGGCCAAGCGCCTCCTGGAGGCCACCATCGAGCCTCTCTCGTGGCTCGAACTCGTGGACTCGATCCAGCGCCTCGGCGTCGCGTACCACTTCGAGGACGAGATCAAGGAAGGCCTCGACGGCGTCTACGGCGTCGGCGCGCACGCCGGCGACGACCTCTACACGGCGGCCCTCCAGTTCCGCCTCCTCCGCCAGCACGGCTACGGCGTCACCCCGGACATCTTCAACAAGTTCCTGGAGAAGGAGCGCACGTTCAAGGCCTGCACCTCGCTCGACGCCAAGGGCCTCCTCTCGCTCTACGAGGCCTCGCACACGATGATCCACGGCGAGGAAGTCCTGGAGGACGCGAAGGAGTTCTCGGTCAAGCACCTCAACTACCTCATGGGCAACCTCCAGAACAACCTCCGCGAGCAGGTCCAGCACGCGCTGGAGATGCCCCTCCACTGGCGCATGCCTCGCCTGGAGGCGAAGCACTACATCGACGTCAACGGCCGCTCGGACGAGCGCAACATGGTCCTCCTCGAACTCGCGCGCCTCGACTTCAACTTCGTCCAGTCGAAGCACCAAGAGGAGCTGAAGGAAGTCTCGCGCTGGTGGCGCGACCTCGGCCTCGCGAAGAAGCTCGGCTTCTCGCGCGACCGCCTCGTCGAGAACTACCTCTGGGCGGTCGGCATCGCCCCCGAGCCCAAGTTCTCGAACTGCCGCAAGGGCCTCACGAAGCTCATCTCGATCCTCACCGTCATCGACGACATCTACGACGTCTACGGCTCGCTCGACGAGCTGGAGCTGTTCACGGAGGCCGTCAAGCGCTGGGACATCGAGGCGCTGGAGACGCTCCCTGAGTACATGAAGATCTGCTACCTCGCCCTCTTCAACTTCGTCCACGAGGTCAGCTACGACACCCTCAAGGACTACGGCTGGAACATCCTCCCGTTCATCCGCGAGGAGTGGGAGCGCCTCTGCATGTCGTACCTCGTCGAGGCCGAGTGGTTCGGCAACGGCAACAAGCCGGCGCTCGACGAGTACCTCCGCAACGGCTGGATCTCGGTCGGCGGCCCCGTCGCGATGGTCCACGCCTACTTCCTCCAGGGCCGCCCTATCCGCAAGGACTCGATCAACTTCCTCGACCACGGCTCGGAGCTGATCTACTGGTCGTCGGTCGCCACGCGCCTCAACGACGACCTCGGCACCTCGAAGGCCGAGATGAAGCGCGGCGACGTCCCCAAGGCCGTCGAGTGCTACATGATCCAGACCGGCGAGTCGTACGAGGACGCGCGCGAGCACATCCAGGGCCTCGTCCGCGACTGCTGGAAGAAGATGAACGAGGAGTGCCTCAAGTGCTGCCTCCCCAAGTCGTACGTCGAGACGGTCCTCAACATGGTCCGCACCGCGCAGTGCATCTACCAGCACGGCGACGGCATCGGCACCTCGACGGGCGTCACCCAGGACCGCGTCATCTCGCTCATCTGCGAGCCGGTCCCCTCGCAGTGGCCGTGA

MHNALQIARRSANYHPSIWDPQYIESLKSPYGDECFGTRLEKLKFEAKRLLEATIEPLSWLELVDSIQRLGVAYHFEDEIKEGLDGVYGVGAHAGDDLYTAALQFRLLRQHGYGVTPDIFNKFLEKERTFKACTSLDAKGLLSLYEASHTMIHGEEVLEDAKEFSVKHLNYLMGNLQNNLREQVQHALEMPLHWRMPRLEAKHYIDVNGRSDERNMVLLELARLDFNFVQSKHQEELKEVSRWWRDLGLAKKLGFSRDRLVENYLWAVGIAPEPKFSNCRKGLTKLISILTVIDDIYDVYGSLDELELFTEAVKRWDIEALETLPEYMKICYLALFNFVHEVSYDTLKDYGWNILPFIREEWERLCMSYLVEAEWFGNGNKPALDEYLRNGWISVGGPVAMVHAYFLQGRPIRKDSINFLDHGSELIYWSSVATRLNDDLGTSKAEMKRGDVPKAVECYMIQTGESYEDAREHIQGLVRDCWKKMNEECLKCCLPKSYVETVLNMVRTAQCIYQHGDGIGTSTGVTQDRVISLICEPVPSQWP

*ag10*

ATGCTCACGACCGTCTCGCACCGCGACGACAACGGCGGCGGCGTCCTCCAGCGCCGAATCGCCGACCACCACCCGAACCTCTGGGAGGACGACTTCATCCAGTCGCTCTCGTCGCCCTACGGCGGCTCGTCGTACTCGGAGCGCGCGGAGACGGTCGTCGAGGAAGTCAAGGAGATGTTCAACTCGATCCCGAACAACCGCGAGCTGTTCGGCTCGCAGAACGACCTCCTCACCCGCCTCTGGATGGTGGACTCGATCGAGCGCCTCGGCATCGACCGCCACTTCCAGAACGAGATCCGCGTCGCGCTCGACTACGTCTACTCGTACTGGAAGGAGAAGGAAGGCATCGGCTGCGGACGCGACTCGACCTTCCCCGACCTCAACTCGACCGCGCTCGCCCTCCGCACCCTCCGCCTCCACGGCTACAACGTCTCGTCGGACGTCCTGGAGTACTTCAAGGACGAGAAGGGCCACTTCGCCTGCCCCGCCATCCTCACCGAGGGCCAGATCACCCGCTCGGTCCTCAACCTCTACCGCGCGTCGCTCGTCGCCTTCCCCGGCGAGAAGGTCATGGAGGAAGCGGAGATCTTCTCGGCCTCGTACCTCAAGAAGGTCCTCCAGAAGATCCCCGTCTCGAACCTCTCGGGCGAGATCGAGTACGTCCTGGAGTACGGCTGGCACACCAACCTCCCTCGCCTGGAGGCCCGCAACTACATCGAGGTCTACGAGCAGTCGGGCTACGAGTCGCTCAACGAGATGCCGTACATGAACATGAAGAAGCTCCTCCAGCTCGCCAAGCTGGAGTTCAACATCTTCCACTCGCTCCAGCTCCGCGAACTCCAGTCGATCTCGCGCTGGTGGAAGGAGTCGGGCTCGTCGCAGCTCACGTTCACCCGCCACCGCCACGTCGAGTACTACACGATGGCGTCGTGCATCTCGATGCTCCCGAAGCACTCGGCCTTCCGCATGGAGTTCGTCAAGGTCTGCCACCTCGTCACGGTCCTCGACGACATCTACGACACGTTCGGCACCATGAACGAACTCCAGCTCTTCACCGACGCGATCAAGCGCTGGGACCTCTCGACGACCCGCTGGCTCCCTGAGTACATGAAGGGCGTCTACATGGACCTCTACCAGTGCATCAACGAGATGGTCGAGGAAGCGGAGAAGACGCAGGGCCGCGACATGCTCAACTACATCCAGAACGCCTGGGAGGCCCTCTTCGACACGTTCATGCAAGAGGCGAAGTGGATCTCGTCGTCGTACCTCCCCACCTTCGAGGAGTACCTCAAGAACGCCAAGGTCAGCTCGGGCTCGCGCATCGCCACCCTCCAGCCCATCCTCACCCTCGACGTCCCCCTCCCCGACTACATCCTCCAGGAGATCGACTACCCCTCGCGCTTCAACGAGCTGGCGTCGTCGATCCTCCGCCTCCGCGGCGACACCCGCTGCTACAAGGCCGACCGCGCGCGCGGCGAGGAAGCGTCGGCCATCTCGTGCTACATGAAGGACCACCCCGGCTCGATCGAGGAAGACGCGCTCAACCACATCAACGCGATGATCTCGGACGCCATCCGCGAGCTGAACTGGGAGCTGCTCCGCCCGGACTCGAAGTCGCCCATCTCGTCGAAGAAGCACGCCTTCGACATCACGCGCGCCTTCCACCACGTCTACAAGTACCGCGACGGCTACACCGTCTCGAACAACGAGACGAAGAACCTCGTCATGAAGACCGTCCTGGAGCCCCTCGCCCTCTGA

MLTTVSHRDDNGGGVLQRRIADHHPNLWEDDFIQSLSSPYGGSSYSERAETVVEEVKEMFNSIPNNRELFGSQNDLLTRLWMVDSIERLGIDRHFQNEIRVALDYVYSYWKEKEGIGCGRDSTFPDLNSTALALRTLRLHGYNVSSDVLEYFKDEKGHFACPAILTEGQITRSVLNLYRASLVAFPGEKVMEEAEIFSASYLKKVLQKIPVSNLSGEIEYVLEYGWHTNLPRLEARNYIEVYEQSGYESLNEMPYMNMKKLLQLAKLEFNIFHSLQLRELQSISRWWKESGSSQLTFTRHRHVEYYTMASCISMLPKHSAFRMEFVKVCHLVTVLDDIYDTFGTMNELQLFTDAIKRWDLSTTRWLPEYMKGVYMDLYQCINEMVEEAEKTQGRDMLNYIQNAWEALFDTFMQEAKWISSSYLPTFEEYLKNAKVSSGSRIATLQPILTLDVPLPDYILQEIDYPSRFNELASSILRLRGDTRCYKADRARGEEASAISCYMKDHPGSIEEDALNHINAMISDAIRELNWELLRPDSKSPISSKKHAFDITRAFHHVYKYRDGYTVSNNETKNLVMKTVLEPLAL

*PT30*

ATGTGCCGCGGCGGCAAGTCGATCGCCCCCTCGATGTCGATGTCGTCCACGACCTCCGTCTCGAACGAGGACGGCGTCCCCCGCCGAATCGCCGGCCACCACTCGAACCTCTGGGACGACGACTCGATCGCCTCGCTCTCGACGTCGTACGAGGCCCCCTCGTACCGCAAGCGCGCCGACAAGCTCATCGGCGAGGTCAAGAACATCTTCGACCTCATGTCGGTCGAGGACGGCGTCTTCACCTCGCCCCTCTCGGACCTCCACCACCGCCTCTGGATGGTGGACTCGGTCGAGCGCCTCGGCATCGACCGCCACTTCAAGGACGAGATCAACTCGGCGCTCGACCACGTCTACTCGTACTGGACGGAGAAGGGCATCGGCCGCGGACGCGAGTCGGGCGTCACCGACCTCAACTCGACGGCCCTCGGCCTCCGCACCCTCCGCCTCCACGGCTACACCGTCTCGTCGCACGTCCTCGACCACTTCAAGAACGAGAAGGGCCAGTTCACGTGCTCGGCCATCCAGACGGAGGGCGAGATCCGCGACGTCCTCAACCTCTTCCGCGCGTCGCTCATCGCCTTCCCCGGCGAGAAGATCATGGAGGCGGCCGAGATCTTCTCGACGATGTACCTCAAGGACGCCCTCCAGAAGATCCCGCCCTCGGGCCTCTCGCAGGAGATCGAGTACCTCCTGGAGTTCGGCTGGCACACGAACCTCCCGCGCATGGAGACGCGCATGTACATCGACGTCTTCGGCGAGGACACGACCTTCGAGACGCCCTACCTCATCCGCGAGAAGCTCCTCGAACTCGCGAAGCTGGAGTTCAACATCTTCCACTCGCTCGTCAAGCGCGAACTCCAGTCGCTCTCGCGCTGGTGGAAGGACTACGGCTTCCCGGAGATCACGTTCTCGCGCCACCGCCACGTCGAGTACTACACCCTCGCGGCCTGCATCGCCAACGACCCTAAGCACTCGGCCTTCCGCCTCGGCTTCGGCAAGATCTCGCACATGATCACGATCCTCGACGACATCTACGACACGTTCGGCACGATGGAGGAGCTGAAGCTCCTCACCGCGGCCTTCAAGCGCTGGGACCCGTCGTCGATCGAGTGCCTCCCTGACTACATGAAGGGCGTCTACATGGCGGTCTACGACAACATCAACGAGATGGCGCGCGAGGCCCAGAAGATCCAGGGCTGGGACACCGTCTCGTACGCGCGCAAGTCGTGGGAGGCGTTCATCGGCGCCTACATCCAAGAGGCGAAGTGGATCTCGTCGGGCTACCTCCCGACGTTCGACGAGTACCTGGAGAACGGCAAGGTCAGCTTCGGCTCGCGCATCACGACGCTGGAGCCGATGCTCACCCTCGGCTTCCCTCTCCCGCCCCGCATCCTCCAGGAGATCGACTTCCCCTCGAAGTTCAACGACCTCATCTGCGCCATCCTCCGCCTCAAGGGCGACACCCAGTGCTACAAGGCCGACCGCGCGCGCGGCGAGGAAGCGTCGGCCGTCTCGTGCTACATGAAGGACCACCCCGGCATCACGGAGGAAGACGCGGTCAACCAGGTCAACGCGATGGTGGACAACCTCACCAAGGAGCTGAACTGGGAGCTGCTCCGCCCGGACTCGGGCGTCCCCATCTCGTACAAGAAGGTCGCGTTCGACATCTGCCGCGTCTTCCACTACGGCTACAAGTACCGCGACGGCTTCTCGGTCGCCTCGATCGAGATCAAGAACCTCGTCACGCGCACGGTCGTCGAGACGGTCCCGCTCTGA

MCRGGKSIAPSMSMSSTTSVSNEDGVPRRIAGHHSNLWDDDSIASLSTSYEAPSYRKRADKLIGEVKNIFDLMSVEDGVFTSPLSDLHHRLWMVDSVERLGIDRHFKDEINSALDHVYSYWTEKGIGRGRESGVTDLNSTALGLRTLRLHGYTVSSHVLDHFKNEKGQFTCSAIQTEGEIRDVLNLFRASLIAFPGEKIMEAAEIFSTMYLKDALQKIPPSGLSQEIEYLLEFGWHTNLPRMETRMYIDVFGEDTTFETPYLIREKLLELAKLEFNIFHSLVKRELQSLSRWWKDYGFPEITFSRHRHVEYYTLAACIANDPKHSAFRLGFGKISHMITILDDIYDTFGTMEELKLLTAAFKRWDPSSIECLPDYMKGVYMAVYDNINEMAREAQKIQGWDTVSYARKSWEAFIGAYIQEAKWISSGYLPTFDEYLENGKVSFGSRITTLEPMLTLGFPLPPRILQEIDFPSKFNDLICAILRLKGDTQCYKADRARGEEASAVSCYMKDHPGITEEDAVNQVNAMVDNLTKELNWELLRPDSGVPISYKKVAFDICRVFHYGYKYRDGFSVASIEIKNLVTRTVVETVPL

*AG3.18*

ATGTCGCGCCGCGGCAAGTCGATCACCCCCTCGATCTCGATGTCGTCCACGACGGTCGTCACGGACGACGGCGTCCGCCGCCGCATGGGCGACTTCCACTCGAACCTCTGGGACGACGACGTCATCCAGTCGCTCCCCACGGCGTACGAGGAGAAGTCGTACCTGGAGCGCGCCGAGAAGCTCATCGGCGAGGTCAAGAACATGTTCAACTCGATGTCGCTGGAGGACGGCGAGCTGATGTCGCCGCTCAACGACCTCATCCAGCGCCTCTGGATCGTGGACTCGCTGGAGCGCCTCGGCATCCACCGCCACTTCAAGGACGAGATCAAGTCGGCGCTCGACTACGTCTACTCGTACTGGGGCGAGAACGGCATCGGCTGCGGACGCGAGTCGGTCGTCACCGACCTCAACTCGACGGCCCTCGGCCTCCGCACCCTCCGCCTCCACGGCTACCCCGTCTCGTCGGACGTCTTCAAGGCCTTCAAGGGCCAGAACGGCCAGTTCTCGTGCTCGGAGAACATCCAGACCGACGAGGAGATCCGCGGCGTCCTCAACCTCTTCCGCGCGTCGCTCATCGCCTTCCCCGGCGAGAAGATCATGGACGAGGCGGAGATCTTCTCGACGAAGTACCTCAAGGAAGCGCTCCAGAAGATCCCCGTCTCGTCGCTCTCGCGCGAGATCGGCGACGTCCTGGAGTACGGCTGGCACACCTACCTCCCTCGCCTGGAGGCCCGCAACTACATCCAGGTCTTCGGCCAGGACACGGAGAACACCAAGTCGTACGTCAAGTCGAAGAAGCTCCTCGAACTCGCGAAGCTGGAGTTCAACATCTTCCAGTCGCTCCAGAAGCGCGAGCTGGAGTCGCTCGTCCGCTGGTGGAAGGAGTCGGGCTTCCCGGAGATGACGTTCTGCCGCCACCGCCACGTCGAGTACTACACCCTCGCGTCGTGCATCGCCTTCGAGCCTCAGCACTCGGGCTTCCGCCTCGGCTTCGCCAAGACGTGCCACCTCATCACCGTCCTCGACGACATGTACGACACGTTCGGCACCGTGGACGAGCTGGAGCTGTTCACGGCGACCATGAAGCGCTGGGACCCGTCGTCGATCGACTGCCTCCCTGAGTACATGAAGGGCGTCTACATCGCCGTCTACGACACCGTCAACGAGATGGCGCGCGAGGCGGAGGAAGCGCAGGGCCGCGACACCCTCACCTACGCCCGCGAGGCGTGGGAGGCCTACATCGACTCGTACATGCAAGAGGCGCGCTGGATCGCCACCGGCTACCTCCCCTCGTTCGACGAGTACTACGAGAACGGCAAGGTCAGCTGCGGCCACCGCATCTCGGCCCTCCAGCCGATCCTCACGATGGACATCCCGTTCCCCGACCACATCCTCAAGGAAGTGGACTTCCCCTCGAAGCTCAACGACCTCGCGTGCGCCATCCTCCGCCTCCGCGGCGACACGCGCTGCTACAAGGCCGACCGCGCGCGCGGCGAGGAAGCGTCGTCGATCTCGTGCTACATGAAGGACAACCCCGGCGTCTCGGAGGAAGACGCGCTCGACCACATCAACGCCATGATCTCGGACGTCATCAAGGGCCTCAACTGGGAGCTGCTCAAGCCGGACATCAACGTCCCCATCTCGGCGAAGAAGCACGCCTTCGACATCGCTCGCGCGTTCCACTACGGCTACAAGTACCGCGACGGCTACTCGGTCGCGAACGTCGAGACGAAGTCGCTCGTCACGCGCACCCTCCTGGAGTCGGTCCCGCTCTGA

MSRRGKSITPSISMSSTTVVTDDGVRRRMGDFHSNLWDDDVIQSLPTAYEEKSYLERAEKLIGEVKNMFNSMSLEDGELMSPLNDLIQRLWIVDSLERLGIHRHFKDEIKSALDYVYSYWGENGIGCGRESVVTDLNSTALGLRTLRLHGYPVSSDVFKAFKGQNGQFSCSENIQTDEEIRGVLNLFRASLIAFPGEKIMDEAEIFSTKYLKEALQKIPVSSLSREIGDVLEYGWHTYLPRLEARNYIQVFGQDTENTKSYVKSKKLLELAKLEFNIFQSLQKRELESLVRWWKESGFPEMTFCRHRHVEYYTLASCIAFEPQHSGFRLGFAKTCHLITVLDDMYDTFGTVDELELFTATMKRWDPSSIDCLPEYMKGVYIAVYDTVNEMAREAEEAQGRDTLTYAREAWEAYIDSYMQEARWIATGYLPSFDEYYENGKVSCGHRISALQPILTMDIPFPDHILKEVDFPSKLNDLACAILRLRGDTRCYKADRARGEEASSISCYMKDNPGVSEEDALDHINAMISDVIKGLNWELLKPDINVPISAKKHAFDIARAFHYGYKYRDGYSVANVETKSLVTRTLLESVPL

*amaOc15*

ATGGCGGAGCTGCCGATGGACTACGAGGGCAAGATCAAGGAGACGCGCCACCTCCTCCACCTCAAGGGCGAGAACGACCCCATCGAGTCGCTCATCTTCGTGGACGCCACCCTCCGCCTCGGCGTCAACCACCACTTCCAGAAGGAGATCGAGGAGATCCTCCGCAAGTCGTACGCCACCATGAAGTCGCCGATCATCTGCGAGTACCACACGCTCCACGAGGTCAGCCTCTTCTTCCGCCTCATGCGCCAGCACGGCCGCTACGTCTCGGCCGACGTCTTCAACAACTTCAAGGGCGAGTCGGGCCGCTTCAAGGAAGAGCTGAAGCGCGACACCCGCGGCCTCGTCGAGCTGTACGAGGCCGCCCAGCTCTCGTTCGAGGGCGAGCGCATCCTCGACGAGGCCGAGAACTTCTCGCGCCAGATCCTCCACGGCAACCTCGCGGGCATGGAGGACAACCTCCGCCGATCGGTCGGCAACAAGCTCCGCTACCCCTTCCACACCTCGATCGCCCGCTTCACGGGCCGCAACTACGACGACGACCTCGGCGGCATGTACGAGTGGGGCAAGACCCTCCGCGAGCTGGCGCTCATGGACCTCCAGGTCGAGCGCTCGGTCTACCAAGAGGAGCTGCTCCAGGTCAGCAAGTGGTGGAACGAGCTGGGCCTCTACAAGAAGCTCAACCTCGCGCGCAACCGCCCGTTCGAGTTCTACACCTGGTCGATGGTCATCCTCGCCGACTACATCAACCTCTCGGAGCAGCGCGTCGAGCTGACGAAGTCGGTCGCGTTCATCTACCTCATCGACGACATCTTCGACGTCTACGGCACCCTCGACGAGCTGATCATCTTCACGGAGGCGGTCAACAAGTGGGACTACTCGGCCACCGACACCCTCCCTGAGAACATGAAGATGTGCTGCATGACGCTCCTCGACACCATCAACGGCACGTCGCAGAAGATCTACGAGAAGCACGGCTACAACCCGATCGACTCGCTCAAGACGACCTGGAAGTCGCTCTGCTCGGCGTTCCTCGTCGAGGCCAAGTGGTCGGCCTCGGGCTCGCTCCCTTCGGCCAACGAGTACCTGGAGAACGAGAAGGTCAGCTCGGGCGTCTACGTCGTCCTCGTCCACCTCTTCTGCCTCATGGGCCTCGGCGGCACCTCGCGCGGCTCGATCGAGCTGAACGACACGCAGGAGCTGATGTCGTCGATCGCGATCATCTTCCGCCTCTGGAACGACCTCGGCTCGGCCAAGAACGAGCACCAGAACGGCAAGGACGGCTCGTACCTCAACTGCTACAAGAAGGAGCACATCAACCTCACCGCGGCCCAGGCCCACGAGCACGCGCTCGAACTCGTCGCGATCGAGTGGAAGCGCCTCAACAAGGAGTCGTTCAACCTCAACCACGACTCCGTCTCGTCGTTCAAGCAGGCCGCCCTCAACCTCGCCCGCATGGTCCCGCTCATGTACTCGTACGACCACAACCAGCGCGGCCCGGTCCTGGAGGAGTACGTCAAGTTCATGCTCTCGGACTGA

MAELPMDYEGKIKETRHLLHLKGENDPIESLIFVDATLRLGVNHHFQKEIEEILRKSYATMKSPIICEYHTLHEVSLFFRLMRQHGRYVSADVFNNFKGESGRFKEELKRDTRGLVELYEAAQLSFEGERILDEAENFSRQILHGNLAGMEDNLRRSVGNKLRYPFHTSIARFTGRNYDDDLGGMYEWGKTLRELALMDLQVERSVYQEELLQVSKWWNELGLYKKLNLARNRPFEFYTWSMVILADYINLSEQRVELTKSVAFIYLIDDIFDVYGTLDELIIFTEAVNKWDYSATDTLPENMKMCCMTLLDTINGTSQKIYEKHGYNPIDSLKTTWKSLCSAFLVEAKWSASGSLPSANEYLENEKVSSGVYVVLVHLFCLMGLGGTSRGSIELNDTQELMSSIAIIFRLWNDLGSAKNEHQNGKDGSYLNCYKKEHINLTAAQAHEHALELVAIEWKRLNKESFNLNHDSVSSFKQAALNLARMVPLMYSYDHNQRGPVLEEYVKFMLSD

*ama1e20*

ATGATCCCCGGCGACGTCGGCTCGACCCCGCCCCCGTCGAAGCTCCACCAGGCGCTCTGCCTCAACGAGCACTCGCTCTCGTGCATGGCCGAGCTGCCGATGGACTACGAGGGCAAGATCAAGGAGACGCGCCACCTCCTCCACCTCAAGGGCGAGAACGACCCGATCGAGTCGCTCATCTTCGTGGACGCCACCCTCCGCCTCGGCGTCAACCACCACTTCCAGAAGGAGATCGAGGAGATCCTCCGCAAGTCGTACGCCACCATGAAGTCGCCGATCATCTGCGAGTACCACACGCTCCACGAGGTCAGCCTCTTCTTCCGCCTCATGCGCCAGCACGGCCGCTACGTCTCGGCGGACGTCTTCAACAACTTCAAGGGCGAGTCGGGCCGCTTCAAGGAAGAGCTGAAGCGCGACACCCGCGGCCTCGTCGAGCTGTACGAGGCCGCCCAGCTCTCGTTCGAGGGCGAGCGCATCCTCGACGAGGCCGAGAACTTCTCGCGCCAGATCCTCCACGGCAACCTCGCGGGCATGGAGGACAACCTCCGCCGATCGGTCGGCAACAAGCTCCGCTACCCCTTCCACACCTCGATCGCCCGCTTCACGGGCCGCAACTACGACGACGACCTCGGCGGCATGTACGAGTGGGGCAAGACCCTCCGCGAGCTGGCGCTCATGGACCTCCAGGTCGAGCGCTCGGTCTACCAAGAGGAGCTGCTCCAGGTCAGCAAGTGGTGGAACGAGCTGGGCCTCTACAAGAAGCTCAACCTCGCGCGCAACCGCCCCTTCGAGTTCTACACCTGGTCGATGGTCATCCTCGCCGACTACATCAACCTCTCGGAGCAGCGCGTCGAGCTGACGAAGTCGGTCGCCTTCATCTACCTCATCGACGACATCTTCGACGTCTACGGCACCCTCGACGAGCTGATCATCTTCACGGAGGCGGTCAACAAGTGGGACTACTCGGCCACCGACACCCTCCCCGAGAACATGAAGATGTGCTGCATGACGCTCCTCGACACCATCAACGGCACGTCGCAGAAGATCTACGAGAAGCACGGCTACAACCCCATCGACTCGCTCAAGACGACCTGGAAGTCGCTCTGCTCGGCGTTCCTCGTCGAGGCCAAGTGGTCGGCCTCGGGCTCGCTCCCCTCGGCCAACGAGTACCTGGAGAACGAGAAGGTCAGCTCGGGCGTCTACGTCGTCCTCGTCCACCTCTTCTGCCTCATGGGCCTCGGCGGCACCTCGCGCGGCTCGATCGAGCTGAACGACACGCAGGAGCTGATGTCGTCGATCGCGATCATCTTCCGCCTCTGGAACGACCTCGGCTCGGCCAAGAACGAGCACCAGAACGGCAAGGACGGCTCGTACCTCAACTGCTACAAGAAGGAGCACATCAACCTCACGGCGGCCCAGGCCCACGAGCACGCGCTCGAACTCGTCGCGATCGAGTGGAAGCGCCTCAACAAGGAGTCGTTCAACCTCAACCACGACTCCGTCTCGTCGTTCAAGCAGGCCGCCCTCAACCTCGCCCGCATGGTCCCCCTCATGTACTCGTACGACCACAACCAGCGCGGCCCCGTCCTGGAGGAGTACGTCAAGTTCATGCTCTCGGACTGA

MIPGDVGSTPPPSKLHQALCLNEHSLSCMAELPMDYEGKIKETRHLLHLKGENDPIESLIFVDATLRLGVNHHFQKEIEEILRKSYATMKSPIICEYHTLHEVSLFFRLMRQHGRYVSADVFNNFKGESGRFKEELKRDTRGLVELYEAAQLSFEGERILDEAENFSRQILHGNLAGMEDNLRRSVGNKLRYPFHTSIARFTGRNYDDDLGGMYEWGKTLRELALMDLQVERSVYQEELLQVSKWWNELGLYKKLNLARNRPFEFYTWSMVILADYINLSEQRVELTKSVAFIYLIDDIFDVYGTLDELIIFTEAVNKWDYSATDTLPENMKMCCMTLLDTINGTSQKIYEKHGYNPIDSLKTTWKSLCSAFLVEAKWSASGSLPSANEYLENEKVSSGVYVVLVHLFCLMGLGGTSRGSIELNDTQELMSSIAIIFRLWNDLGSAKNEHQNGKDGSYLNCYKKEHINLTAAQAHEHALELVAIEWKRLNKESFNLNHDSVSSFKQAALNLARMVPLMYSYDHNQRGPVLEEYVKFMLSD

*AG2.2*

ATGGCGTCGCTCGCCACGGCGGCCCCCGACGACGGCGTCCAGCGCCGAATCGGCGACTACCACTCGAACATCTGGGACGACGACTTCATCCAGTCGCTCTCGACCCCGTACGGCGAGCCTTCGTACCAGGAGCGCGCCGAGCGCCTCATCGTCGAGGTCAAGAAGATCTTCAACTCGATGTACCTCGACGACGGCCGCCTCATGTCGTCGTTCAACGACCTCATGCAGCGCCTCTGGATCGTGGACTCGGTCGAGCGCCTCGGCATCGCCCGCCACTTCAAGAACGAGATCACGTCGGCCCTCGACTACGTCTTCCGCTACTGGGAGGAGAACGGCATCGGCTGCGGACGCGACTCGATCGTCACGGACCTCAACTCGACGGCCCTCGGCTTCCGCACCCTCCGCCTCCACGGCTACACCGTCTCGCCGGAGGTCCTCAAGGCCTTCCAGGACCAGAACGGCCAGTTCGTCTGCTCGCCCGGCCAGACGGAGGGCGAGATCCGCTCGGTCCTCAACCTCTACCGCGCGTCGCTCATCGCCTTCCCCGGCGAGAAGGTCATGGAGGAAGCGGAGATCTTCTCGACGCGCTACCTCAAGGAAGCGCTCCAGAAGATCCCCGTCTCGGCCCTCTCGCAGGAGATCAAGTTCGTCATGGAGTACGGCTGGCACACCAACCTCCCTCGCCTGGAGGCCCGCAACTACATCGACACGCTGGAGAAGGACACCTCGGCGTGGCTCAACAAGAACGCCGGCAAGAAGCTCCTCGAACTCGCGAAGCTGGAGTTCAACATCTTCAACTCGCTCCAGCAAAAGGAACTCCAGTACCTCCTCCGCTGGTGGAAGGAGTCGGACCTCCCCAAGCTCACCTTCGCCCGCCACCGCCACGTCGAGTTCTACACCCTCGCGTCGTGCATCGCCATCGACCCTAAGCACTCGGCGTTCCGCCTCGGCTTCGCCAAGATGTGCCACCTCGTCACGGTCCTCGACGACATCTACGACACGTTCGGCACCATCGACGAGCTGGAGCTGTTCACGTCGGCCATCAAGCGCTGGAACTCGTCGGAGATCGAGCACCTCCCGGAGTACATGAAGTGCGTCTACATGGTCGTCTTCGAGACGGTCAACGAGCTGACCCGCGAGGCCGAGAAGACGCAGGGCCGCAACACCCTCAACTACGTCCGCAAGGCGTGGGAGGCCTACTTCGACTCGTACATGGAGGAAGCGAAGTGGATCTCGAACGGCTACCTCCCGATGTTCGAGGAGTACCACGAGAACGGCAAGGTCAGCTCGGCCTACCGCGTCGCCACCCTCCAGCCCATCCTCACCCTCAACGCCTGGCTCCCCGACTACATCCTCAAGGGCATCGACTTCCCCTCGCGCTTCAACGACCTCGCCTCGTCGTTCCTCCGCCTCCGCGGCGACACGCGCTGCTACAAGGCCGACCGCGACCGCGGCGAGGAAGCGTCGTGCATCTCGTGCTACATGAAGGACAACCCCGGCTCGACGGAGGAAGACGCGCTCAACCACATCAACGCGATGGTCAACGACATCATCAAGGAGCTGAACTGGGAGCTGCTCCGCTCCAACGACAACATCCCCATGCTCGCGAAGAAGCACGCCTTCGACATCACGCGCGCGCTCCACCACCTCTACATCTACCGCGACGGCTTCTCGGTCGCCAACAAGGAGACGAAGAAGCTCGTCATGGAGACGCTCCTGGAGTCGATGCTCTTCTGA

MASLATAAPDDGVQRRIGDYHSNIWDDDFIQSLSTPYGEPSYQERAERLIVEVKKIFNSMYLDDGRLMSSFNDLMQRLWIVDSVERLGIARHFKNEITSALDYVFRYWEENGIGCGRDSIVTDLNSTALGFRTLRLHGYTVSPEVLKAFQDQNGQFVCSPGQTEGEIRSVLNLYRASLIAFPGEKVMEEAEIFSTRYLKEALQKIPVSALSQEIKFVMEYGWHTNLPRLEARNYIDTLEKDTSAWLNKNAGKKLLELAKLEFNIFNSLQQKELQYLLRWWKESDLPKLTFARHRHVEFYTLASCIAIDPKHSAFRLGFAKMCHLVTVLDDIYDTFGTIDELELFTSAIKRWNSSEIEHLPEYMKCVYMVVFETVNELTREAEKTQGRNTLNYVRKAWEAYFDSYMEEAKWISNGYLPMFEEYHENGKVSSAYRVATLQPILTLNAWLPDYILKGIDFPSRFNDLASSFLRLRGDTRCYKADRDRGEEASCISCYMKDNPGSTEEDALNHINAMVNDIIKELNWELLRSNDNIPMLAKKHAFDITRALHHLYIYRDGFSVANKETKKLVMETLLESMLF

*PaTPS-Lin*

ATGTGCCGCCTCACCAAGTCGGTCACGCCGTCGATCTCGATGTGCCTCACCACGACCGTCTCGGACGACGGCGTCCAGCGCCGAATCGCCGACCACCACCCGAACCTCTGGGACGACAACTTCATCCAGTCGCTCTCGACCCCCTACGGCGCCACCGCCTACCACGAGCGCGCGCAGAAGCTCATCGGCGAGGTCAAGGTCATCATCAACTCGATCCTCGTCGAGGACGGCGAGCTGATCACGCCGCCCAACGACCTCCTCCAGCGCCTCTCGATCGTGGACTCGATCGAGCGCCTCGGCATCGACCGCCACTTCAAGAACGAGATCAAGTCGGCCCTCGACTACGTCTACTCGTACTGGTCGGAGAAGGGCATCGGCTGCGGACGCGACTCGGTCGTCAACGACCTCAACACGACCGCCCTCGGCCTCCGCACCCTCCGCCTCCACGGCTACCCCGTCTCGTCGGACGTCCTGGAGCAGTTCAAGGACCAGAACGGCCAGTTCGCGTGCTCGGCCATCCAGACGGAGGGCGAGATCAAGACGGTCCTCAACCTCTTCCGCGCCTCGCTCATCGCCTTCCCCGGCGAGAAGGTCATGGAGGAAGCGGAGATCTTCTCGACGATCTACCTCAAGGAAGCGCTCCTCAAGATCCCGGTCTGCTCGCTCTCGCGCGAGATCGCGTACGTCCTGGAGTACGGCTGGCACATGAACCTCCCCCGCCTGGAGGCCCGCAACTACATCGACGTCTTCGGCCAGGACCCCATCTACCTCCGCTCGACCCAGAAGCTCATCGAGCTGGCGAAGCTGGAGTTCAACATCTTCCAGTCGCTCCAGCAAGAGGAGCTGAAGCACGTCTCGCGCTGGTGGAAGGACTCGGGCTTCTCGCAGATGGCCTTCGCCCGCCACCGCCACGTCGAGTACTACACGCTCGCGTCGTGCATCGACATCTACCCGCAGCACTCGTCGTTCCGCCTCGGCTTCGCGAAGATCGCCCACCTCGGCACCGTCCTCGACGACATCTACGACACCTTCGGCACGATGGACGAGCTGGAGCTGTTCACGGCGGCGGTCAAGCGCTGGCACCCCTCGGCGGCCGAGGGCCTCCCTGAGTACATGAAGGGCGTCTACATGATGTTCTACGAGACGGTCAACGAGATGGCGCGCGAGGCGGAGAAGTCGCAGGGCCGCGACACCCTCAACTACGCCCGCCAGGCGCTGGAGGCGTACATCGACTCGTACATGAAGGAAGCGAAGTGGATCTCGTCGGGCTTCCTCCCCACCTTCGAGGAGTACCTCGACAACGGCAAGGTCAGCTTCGGCTACCGCATCGCCACCCTCCAGCCCATCCTCACCCTCGGCATCCCGTTCCCCCACCACATCCTCCAGGAGATCGACTTCCCCTCGCGCCTCAACGACCTCGCGGGCTCGATCCTCCGCCTCAAGGGCGACATCCACTCGTACCAGGCCGAGCGCTCGCGCGGCGAGGAGTCGTCGTGCATCTCGTGCTACATGAAGGACAACCCCGAGGCCACGGAGGAAGACGCCGTCACGTACATCAACGCGATGGTCAACCGCCTCCTCAAGGAGCTGAACTGGGAGCTGCTCAAGCCGGACAACAACGTCCCCATCACGTCGAAGAAGCACGCCTTCGACATCCTCCGCGCGTTCTACCACCTCTACAAGGACCGCGACGGCTTCTCGGTCGCCCGCAACGAGATCCGCAACCTCGTCATGACCACGGTCATCGAGCACGTCCCCCTCTGA

MCRLTKSVTPSISMCLTTTVSDDGVQRRIADHHPNLWDDNFIQSLSTPYGATAYHERAQKLIGEVKVIINSILVEDGELITPPNDLLQRLSIVDSIERLGIDRHFKNEIKSALDYVYSYWSEKGIGCGRDSVVNDLNTTALGLRTLRLHGYPVSSDVLEQFKDQNGQFACSAIQTEGEIKTVLNLFRASLIAFPGEKVMEEAEIFSTIYLKEALLKIPVCSLSREIAYVLEYGWHMNLPRLEARNYIDVFGQDPIYLRSTQKLIELAKLEFNIFQSLQQEELKHVSRWWKDSGFSQMAFARHRHVEYYTLASCIDIYPQHSSFRLGFAKIAHLGTVLDDIYDTFGTMDELELFTAAVKRWHPSAAEGLPEYMKGVYMMFYETVNEMAREAEKSQGRDTLNYARQALEAYIDSYMKEAKWISSGFLPTFEEYLDNGKVSFGYRIATLQPILTLGIPFPHHILQEIDFPSRLNDLAGSILRLKGDIHSYQAERSRGEESSCISCYMKDNPEATEEDAVTYINAMVNRLLKELNWELLKPDNNVPITSKKHAFDILRAFYHLYKDRDGFSVARNEIRNLVMTTVIEHVPL

*RlemTPS2*

ATGGGCTTCGTCCCGCCCATCACGCGCGTCCAGTACCACGTCGCCGCCTCGACGACCCCGATCAAGCCCGTGGACCAGACGATCATCCGCCGATCGGCCGACTACGGCCCCACCATCTGGTCGTTCGACTACATCCAGTCGCTCGACTCGAAGTACAAGGGCGAGTCGTACGCCCGCCAGTCGGAGAAGCTCAAGGAGCAGGTCAGCGCGATGCTCCAGCAGGACGACAAGGTCGTGGACCTCGACCCGCTCCACCAGCTCGAACTCATCGACAACCTCCACCGCCTCGGCGTCTCGTACCACTTCGAGGACGAGATCAAGCGCACCCTCGACCGCATCCACAACAAGAACACGAACAAGTCGCTCTACGCGACCGCCCTCAAGTTCCGCATCCTCCGCCAGCACGGCTACAACACGCCGGTCAAGGAGACGTTCTCGCGCTTCATGGACGAGAAGGGCATCTTCAAGCTCTCGTCGCACTCGGACGACTGCAAGGGCATGCTCGCGCTCTACGAGGCGGCCTACCTCCTCGTCGAGGAAGAGTCGTCGATCTTCCGCGACGCGACGTCGTTCACGACCGCCTACCTCAAGGAGTGGGTCATCAAGCACGACAACATCAAGCACGACGACGAGCACCTCTGCACCCTCGTCAACCACGCGCTCGAACTCCCCCTCCACTGGCGCATGCCCCGCCTGGAGGCCCGCTGGTTCATCGACGTCTACGAGAACGGCCCGGACATGTCGCCCATCCTCCTCGAACTCGCGAAGGTGGACTTCAACATCGTCCAGGCCGTCCACCAGGAGAACCTCAAGTACGCCTCGCGCTGGTGGAAGAAGACGGGCCTCGGCGAGAACCTCAACTTCGTCCGCGACCGCATCGTCGAGAACTTCCTCTGGACCGTCGGCGAGAAGTTCGAGCCGCAGTTCGGCTACTTCCGCCGAATGTCCACGATGGTCATCGCGCTCATCACGGCGGTGGACGACGTCTACGACGTCTACGGCACGCTCGACGAGCTGGAGATCTTCACCGACGCGGTCGAGCGCTGGGACGCCACGGCCGTCGAGCAGCTCCCCCACTACATGAAGCTCTGCTTCCACGCCCTCCGCAACTCGATCAACGAGATGACCTTCGACGCGCTCCGCGACCAGGGCGTGGACATCGTCATCTCGTACCTCACCAAGGCGTGGGCCGACATCTGCAAGGCGTACCTCGTCGAGGCCAAGTGGTACAACTCGGGCTACATCCCCTCGCTCCAGGAGTACATGGAGAACGCCTGGATCTCGATCGGCTCGACGGTCATCCTCGTCCACGCCTACACGTTCACCGCCAACCCGATCACCAAGGAAGGCCTGGAGTTCGTCAAGGACTACCCCAACATCATCCGCTGGTCGTCGGTCATCCTCCGCTTCGCGGACGACCTCGGCACGTCGTCGGACGAGCTGAAGCGCGGCGACGTCCACAAGTCGATCCAGTGCTACATGCACGAGGCCGGCGTCTCGGAGGGCGAGGCCCGCGAGCACATCAACGACCTCATCGCGCAGACCTGGATGAAGATGAACCGCGACCGCTTCGGCAACCCGCACTTCGTCTCGGACGTCTTCGTCGGCATCGCGATGAACCTCGCCCGCATGTCGCAGTGCATGTACCAGTTCGGCGACGGCCACGGCTGCGGCGCGCAGGAGATCACCAAGGCCCGCGTCCTCTCGCTCTTCATCGACCCCATCGCGTGA

MGFVPPITRVQYHVAASTTPIKPVDQTIIRRSADYGPTIWSFDYIQSLDSKYKGESYARQSEKLKEQVSAMLQQDDKVVDLDPLHQLELIDNLHRLGVSYHFEDEIKRTLDRIHNKNTNKSLYATALKFRILRQHGYNTPVKETFSRFMDEKGIFKLSSHSDDCKGMLALYEAAYLLVEEESSIFRDATSFTTAYLKEWVIKHDNIKHDDEHLCTLVNHALELPLHWRMPRLEARWFIDVYENGPDMSPILLELAKVDFNIVQAVHQENLKYASRWWKKTGLGENLNFVRDRIVENFLWTVGEKFEPQFGYFRRMSTMVIALITAVDDVYDVYGTLDELEIFTDAVERWDATAVEQLPHYMKLCFHALRNSINEMTFDALRDQGVDIVISYLTKAWADICKAYLVEAKWYNSGYIPSLQEYMENAWISIGSTVILVHAYTFTANPITKEGLEFVKDYPNIIRWSSVILRFADDLGTSSDELKRGDVHKSIQCYMHEAGVSEGEAREHINDLIAQTWMKMNRDRFGNPHFVSDVFVGIAMNLARMSQCMYQFGDGHGCGAQEITKARVLSLFIDPIA

*SabS1*

ATGCCGCTCAACTCGCTCCACAACCTGGAGCGCAAGCCCTCGAAGGCGTGGTCCACGTCGTGCACGGCCCCTGCGGCGCGCCTCCAGGCGTCGTTCTCGCTCCAGCAAGAGGAGCCTCGCCAGATCCGCCGATCGGGCGACTACCAGCCGTCGCTCTGGGACTTCAACTACATCCAGTCGCTCAACACGCCCTACAAGGAGCAGCGCTACGTCAACCGCCAGGCCGAGCTGATCATGCAGGTCCGCATGCTCCTCAAGGTCAAGATGGAGGCGATCCAGCAGCTCGAACTCATCGACGACCTCCAGTACCTCGGCCTCTCGTACTTCTTCCCGGACGAGATCAAGCAGATCCTCTCGTCGATCCACAACGAGCACCGCTACTTCCACAACAACGACCTCTACCTCACGGCCCTCGGCTTCCGCATCCTCCGCCAGCACGGCTTCAACGTCTCGGAGGACGTCTTCGACTGCTTCAAGACGGAGAAGTGCTCGGACTTCAACGCGAACCTCGCCCAGGACACCAAGGGCATGCTCCAGCTCTACGAGGCCTCGTTCCTCCTCCGCGAGGGCGAGGACACGCTCGAACTCGCGCGCCGCTTCTCGACCCGCTCGCTCCGCGAGAAGCTCGACGAGGACGGCGACGAGATCGACGAGGACCTCTCGTCGTGGATCAGGCACTCGCTCGACCTCCCTCTCCACTGGCGCATCCAGGGCCTGGAGGCCCGCTGGTTCCTCGACGCCTACGCGCGCCGCCCTGACATGAACCCCCTCATCTTCAAGCTCGCCAAGCTCAACTTCAACATCGTCCAGGCGACGTACCAAGAGGAGCTGAAGGACGTCTCGCGCTGGTGGAACTCGTCGTGCCTCGCCGAGAAGCTCCCTTTCGTCCGCGACCGCATCGTCGAGTGCTTCTTCTGGGCCATCGGCGCCTTCGAGCCCCACCAGTACTCGTACCAGCGCAAGATGGCGGCCATCATCATCACGTTCGTCACCATCATCGACGACGTCTACGACGTCTACGGCACGCTGGAGGAGCTGGAGCTGTTCACGGACATGATCCGCCGATGGGACAACATCTCGATCTCGCAGCTCCCCTACTACATGCAGGTCTGCTACCTCGCCCTCTACAACTTCGTCTCGGAGCGCGCGTACGACATCCTCAAGGACCAGCACTTCAACTCGATCCCGTACCTCCAGCGCTCGTGGGTCAGCCTCGTCGAGGGCTACCTCAAGGAAGCGTACTGGTACTACAACGGCTACAAGCCGTCGCTGGAGGAGTACCTCAACAACGCGAAGATCTCGATCTCGGCCCCGACGATCATCTCGCAGCTCTACTTCACCCTCGCGAACTCGACGGACGAGACGGTCATCGAGTCGCTCTACGAGTACCACAACATCCTCTACCTCTCGGGCACGATCCTCCGCCTCGCCGACGACCTCGGCACCTCGCAGCACGAGCTGGAGCGCGGCGACGTCCCTAAGGCCATCCAGTGCTACATGAAGGACACCAACGCCTCGGAGCGCGAGGCGGTCGAGCACGTCAAGTTCCTCATCCGCGAGACGTGGAAGGAGATGAACACCGTCACCACGGCCTCGGACTGCCCGTTCACGGACGACCTCGTCGCCGTCGCCACCAACCTCGCTCGCGCGGCCCAGTTCATCTACCTCGACGGCGACGGCCACGGCGTCCAGCACTCGGAGATCCACCAGCAGATGGGCGGCCTCCTCTTCCAGCCGTACGTCTGA

MPLNSLHNLERKPSKAWSTSCTAPAARLQASFSLQQEEPRQIRRSGDYQPSLWDFNYIQSLNTPYKEQRYVNRQAELIMQVRMLLKVKMEAIQQLELIDDLQYLGLSYFFPDEIKQILSSIHNEHRYFHNNDLYLTALGFRILRQHGFNVSEDVFDCFKTEKCSDFNANLAQDTKGMLQLYEASFLLREGEDTLELARRFSTRSLREKLDEDGDEIDEDLSSWIRHSLDLPLHWRIQGLEARWFLDAYARRPDMNPLIFKLAKLNFNIVQATYQEELKDVSRWWNSSCLAEKLPFVRDRIVECFFWAIGAFEPHQYSYQRKMAAIIITFVTIIDDVYDVYGTLEELELFTDMIRRWDNISISQLPYYMQVCYLALYNFVSERAYDILKDQHFNSIPYLQRSWVSLVEGYLKEAYWYYNGYKPSLEEYLNNAKISISAPTIISQLYFTLANSTDETVIESLYEYHNILYLSGTILRLADDLGTSQHELERGDVPKAIQCYMKDTNASEREAVEHVKFLIRETWKEMNTVTTASDCPFTDDLVAVATNLARAAQFIYLDGDGHGVQHSEIHQQMGGLLFQPYV

*PaJF67*

ATGGCGTCGAAGTCCACGTCGCTCACCACCGCCGTCTCGGACGACGGCGTCCAGCGCCGAATCGGCGACCACCACTCGAACCTCTGGGACGACAACTTCATCCAGTCGCTCTCGTCGCCGTACGGCGCGTCGTCGTACGGCGAGCGCGCCGAGCGCCTCATCGGCGAGGTCAAGGAGATCTTCAACTCGCTCTCGCGCACCGACGGCGAACTCGTCTCGCACGTGGACGACCTCCTCCAGCACCTCTCGATGGTGGACAACGTCGAGCGCCTCGGCATCGACCGCCACTTCCAGACCGAGATCAAGGTCAGCCTCGACTACGTCTACTCGTACTGGTCGGAGAAGGGCATCGGCTCGGGCCGCGACATCGTCTGCACCGACCTCAACACCACGGCCCTCGGCTTCCGCATCCTCCGCCTCCACGGCTACACCGTCTTCCCGGACGTCTTCGAGCACTTCAAGGACCAGATGGGCCGCATCGCCTGCTCGGACAACCACACGGAGCGCCAGATCTCGTCGATCCTCAACCTCTTCCGCGCGTCGCTCATCGCCTTCCCCGGCGAGAAGGTCATGGAGGAAGCGGAGATCTTCTCGGCCACGTACCTCAAGGAAGCGCTCCAGACCATCCCCGTCTCGTCGCTCTCGCAGGAGATCCAGTACGTCCTCCAGTACCGCTGGCACTCGAACCTCCCTCGCCTGGAGGCCCGCACGTACATCGACATCCTCCAGGAGAACACCAAGAACCAGATGCTCGACGTCAACACGAAGAAGGTCCTCGAACTCGCGAAGCTGGAGTTCAACATCTTCCACTCGCTCCAGCAGAACGAGCTGAAGTCCGTCTCGCGCTGGTGGAAGGAGTCGGGCTTCCCCGACCTCAACTTCATCCGCCACCGCCACGTCGAGTTCTACACCCTCGTCTCGGGCATCGACATGGAGCCCAAGCACTGCACGTTCCGCCTCTCGTTCGTCAAGATGTGCCACCTCATCACCGTCCTCGACGACATGTACGACACGTTCGGCACCATCGACGAGCTGCGCCTCTTCACGGCGGCCGTCAAGCGCTGGGACCCCTCGACGACGGAGTGCCTCCCTGAGTACATGAAGGGCGTCTACACGGTCCTCTACGAGACGGTCAACGAGATGGCCCAAGAGGCGCAGAAGTCGCAGGGCCGCGACACCCTCTCGTACGTCCGCCAGGCGCTGGAGGCGTACATCGGCGCGTACCACAAGGAAGCGGAGTGGATCTCGTCGGGCTACCTCCCGACCTTCGACGAGTACTTCGAGAACGGCAAGGTCAGCTCGGGCCACCGCATCGCCACCCTCCAGCCGACCTTCATGCTCGACATCCCGTTCCCCCACCACGTCCTCCAGGAGATCGACTTCCCCTCGAAGTTCAACGACTTCGCCTGCTCGATCCTCCGCCTCCGCGGCGACACGCGCTGCTACCAGGCCGACCGCGCGCGCGGCGAGGAAGCGTCGTGCATCTCGTGCTACATGAAGGACAACCCCGGCTCGACGCAAGAGGACGCGCTCAACCACATCAACAACATGATCGAGGAGACGATCAAGAAGCTCAACTGGGAGCTGCTCAAGCCGGACAACAACGTCCCCATCTCGTCGAAGAAGCACGCCTTCGACATCAACCGCGGCCTCCACCACTTCTACAACTACCGCGACGGCTACACGGTCGCCTCGAACGAGACGAAGAACCTCGTCATCAAGACCGTCCTGGAGCCGGTCCCCATGTGA

MASKSTSLTTAVSDDGVQRRIGDHHSNLWDDNFIQSLSSPYGASSYGERAERLIGEVKEIFNSLSRTDGELVSHVDDLLQHLSMVDNVERLGIDRHFQTEIKVSLDYVYSYWSEKGIGSGRDIVCTDLNTTALGFRILRLHGYTVFPDVFEHFKDQMGRIACSDNHTERQISSILNLFRASLIAFPGEKVMEEAEIFSATYLKEALQTIPVSSLSQEIQYVLQYRWHSNLPRLEARTYIDILQENTKNQMLDVNTKKVLELAKLEFNIFHSLQQNELKSVSRWWKESGFPDLNFIRHRHVEFYTLVSGIDMEPKHCTFRLSFVKMCHLITVLDDMYDTFGTIDELRLFTAAVKRWDPSTTECLPEYMKGVYTVLYETVNEMAQEAQKSQGRDTLSYVRQALEAYIGAYHKEAEWISSGYLPTFDEYFENGKVSSGHRIATLQPTFMLDIPFPHHVLQEIDFPSKFNDFACSILRLRGDTRCYQADRARGEEASCISCYMKDNPGSTQEDALNHINNMIEETIKKLNWELLKPDNNVPISSKKHAFDINRGLHHFYNYRDGYTVASNETKNLVIKTVLEPVPM

*TpsB*

ATGCTCCCCACCGACGAGTTCCAGGTCGAGCGCCGATCGGGCAACTACTCGCCCTCGAAGTGGGACGTGGACTACATCCAGTCGCTCCACTCGGACTACAAGGAAGAGCGCCACACGCGCCGCGCGTCGGAGCTGATCATGGAGGTCAAGAAGCTCCTGGAGAAGGAGCCGAACCCCACCCGCCAGCTCGAACTCATCGACGACCTCCAGAAGCTCGGCCTCTCGGACCACTTCAACAACGAGTTCAAGGAGATCCTCAACTCGGTCTACCTCGACAACAAGTACTACCGCAACGGCGCGATGAAGGAAGTCGAGCGCGACCTCTACTCGACGGCGCTCGCCTTCCGCCTCCTCCGCCAGCACGGCTTCCAGGTCGCCCAGGACGTCCTGGAGTGCTTCAAGAACACGAAGGGCGAGTTCGAGCCGTCGCTCTCGGACGACACCCGCGGCCTCCTCCAGCTCTACGAGGCGTCGTTCCTCCTCACGGAGGGCGAGAACACGCTCGAACTCGCGCGCGACTTCACGACCAAGATCCTGGAGGAGAAGCTCCGCAACGACGAGATCGACGACATCAACCTCGTCACGTGGATCAGGCACTCGCTGGAGATCCCCATCCACTGGCGCATCGACCGCGTCAACACCTCGGTCTGGATCGACGTCTACAAGCGCCGCCCTGACATGAACCCCATCGTCCTCGAACTCGCGGTCCTCGACTCGAACATCGTCCAGGCCCAGTACCAAGAGGAGCTGAAGCTCGACCTCCAGTGGTGGCGCAACACGTGCCTCGCCGAGAAGCTCCCCTTCGCCCGCGACCGCCTCGTCGAGTCGTACTTCTGGGGCGTCGGCGTCGTCCAGCCTCGCCAGCACGGCATCGCCCGCATGGCGGTGGACCGCTCGATCGCGCTCATCACCGTCATCGACGACGTCTACGACGTCTACGGCACGCTGGAGGAGCTGGAGCAGTTCACCGAGGCCATCCGCCGATGGGACATCTCGTCGATCGACCAGCTCCCGTCGTACATGCAGCTCTGCTTCCTCGCGCTCGACAACTTCATCAACGACATCGCCTACGACGTCCTCAAGGAGCAGGGCTTCAACATCATCCCGTACCTCCGCAAGTCGTGGACGGACATGATCGAGGGCTTCCTCCTGGAGGCGAAGTGGTATCACAACGGCCACAAGCCCAAGCTGGAGGAGTACCTGGAGAACGGCTGGCGCTCGATCGGCTCGACGGTCGTCCTCACCCACGCCTTCTTCGGCGTCACGCACTCGCTCACCAAGGAGAACATCGACCAGTTCTTCGGCTACCACGAGATCGTCCGCCTCTCGTCGATGCTCCTCCGCCTCGCCGACGACCTCGGCACCTCGACGGACGAGGTCAGCCGCGGCGACGTCCCGAAGGCGATCCAGTGCTACATGAACGACAACATCGGCGCCTCGGAGGCCGAGGCCCGCGAGCACGTCAAGTGGTGCATCTGGGAGACGTGGAAGAAGATGAACAAGGTCCGCGTCGCGCGCGACACCCCCTTCTCGCAGGACTTCATCGTCTGCGCGATGGGCATGGGCCGCATGGGCCAGTACATGTACCACTACGGCGACGGCCACGGCATCCAGCACTCGATCATCCACCAGCAGATGTCCACGTGCCTCTTCCACCCGTCGTCGTCGAACTGA

MLPTDEFQVERRSGNYSPSKWDVDYIQSLHSDYKEERHTRRASELIMEVKKLLEKEPNPTRQLELIDDLQKLGLSDHFNNEFKEILNSVYLDNKYYRNGAMKEVERDLYSTALAFRLLRQHGFQVAQDVLECFKNTKGEFEPSLSDDTRGLLQLYEASFLLTEGENTLELARDFTTKILEEKLRNDEIDDINLVTWIRHSLEIPIHWRIDRVNTSVWIDVYKRRPDMNPIVLELAVLDSNIVQAQYQEELKLDLQWWRNTCLAEKLPFARDRLVESYFWGVGVVQPRQHGIARMAVDRSIALITVIDDVYDVYGTLEELEQFTEAIRRWDISSIDQLPSYMQLCFLALDNFINDIAYDVLKEQGFNIIPYLRKSWTDMIEGFLLEAKWYHNGHKPKLEEYLENGWRSIGSTVVLTHAFFGVTHSLTKENIDQFFGYHEIVRLSSMLLRLADDLGTSTDEVSRGDVPKAIQCYMNDNIGASEAEAREHVKWCIWETWKKMNKVRVARDTPFSQDFIVCAMGMGRMGQYMYHYGDGHGIQHSIIHQQMSTCLFHPSSSN

*LcTPS2*

ATGTCGGCCCCCTCGTACTCGGACCTCGTCCGCCGCCGCTCGGCCAACTACAAGCCTTCGAAGTGGGACTCGAACTTCGTCGAGACGCTGGAGTCGGACTACAAGAAGGAGAACCACGAGATGTACATCGAGAAGCTCATGGGCGACGTCAAGCACCTCATGAAGGAAGTCGTCAACCCGATCGAGAAGATGGAGCTGGTGGACACCATCCAGCGCCTCGGCCTCGGCTACCTCTTCAACAAGGAGATCAAGGAAGTCCTCAACACCATCACGACCTCGAAGGCCACGCTCAAGACCAAGAAGGACCTCCACGCCGTCGCCCTCCAGTTCCGCCTCCTCCGCCAGCACGGCTACGAGGTCAGCCCCGACGCCTTCCACGAGTTCAAGGACGAGAAGGGCGGCTTCAAGGAGTCGCTCTGCATGGACATCAAGGGCATGCTCTGCCTCTACGAGGCGTCGCACCTCTCGTTCCAGGGCGAGGTCGTCCTCGACGAGGCGCGCGAGTTCACCTCGACGCACCTCAAGGCGATCGGCGGCAACATCGACCCGGTCCTCCTCAAGAAGGTCCGCCACTCGCTGGAGATGCCCCTCCACTGGCGCATGCTCCGCCTGGAGGCCCGCTGGTACATCGAGACGTACGACGAGGAAGACCGCAAGAACCCGTCGCTCGCGGAGCTGGCGAAGCACGACTTCAACTCGGTCCAGACCATCTACCAGCGCTCGCTCAAGCGCATGTCGCGCTGGTGGCGCACCTCGGCCTCGGCGAGCGCCTGGAGTTCTCGCGCGACCGCCTCGTCGAGTGCTTCTTCTGGACGACCGGCGTCATCTTCGACCCCCAGTTCGAGCGCTGCCGCGGCGTCCTCACCAAGGTCAACCAGCTCGTCTCGACCATCGACGACGTCTACGACGTCTACGGCTCGCTGGAGGAGCTGGAGCTGTTCACGGACGCGGTGGACCGCTGGGACATCCGCGCGATGGAGCAGCTCCCTGAGTACATGAAGATCTGCTACCTCGCCCTCTACAACACGACCAACGACATCGCGTACGAGGCCCTCAAGGAAGAGGGCCTCGACGTCATCCCGTACCTCAAGAAGGTCTGGACCGACCTCTGCAAGTCGTACATCGTCGAGGCCCGCTGGTACTCGAACGGCTACAAGCCCACGCTGGAGGAGTACCTGGAGAACGCCTGGACCTCGATCGCCGGCCCCGTCGCCCTCGTCCACGCCTACTTCTCGTTCGGCCAGAAGATGCCCTTCGAGGCGCTCAACTACTCGAACACGTCGTCGCTCATCAAGTGGTCGTCGATGATCTTCCGCCTCTGCGACGACCTCGCCACCTCGTCGGACGAGGTCGCGCGCGGCGACGTCCCGAAGTCGATCCAGTGCTACATGTACGAGGCGGGCGTCTCGGAGTCGGTCGCGCGCGACCACATCAAGTACCTCATCGACGAGGCCTGGAAGAAGATGAACGAGTGCCTCGTCTACAACACGCCGTTCCTCCAGCCCCTCATCAACGCCGGCCTCAACCTCGCCCGCATGGCCCACTGCATGTACGAGCGCGGCGACGGCCACGGCTTCTCGAACGAGCTGGACAAGAAGCGCGTCCTCCTCCTCCTCGCGGAGCCCTTCAAGTTCATGTGA

MSAPSYSDLVRRRSANYKPSKWDSNFVETLESDYKKENHEMYIEKLMGDVKHLMKEVVNPIEKMELVDTIQRLGLGYLFNKEIKEVLNTITTSKATLKTKKDLHAVALQFRLLRQHGYEVSPDAFHEFKDEKGGFKESLCMDIKGMLCLYEASHLSFQGEVVLDEAREFTSTHLKAIGGNIDPVLLKKVRHSLEMPLHWRMLRLEARWYIETYDEEDRKNPSLAELAKHDFNSVQTIYQRSLKRMSRWWRDLGLGERLEFSRDRLVECFFWTTGVIFDPQFERCRGVLTKVNQLVSTIDDVYDVYGSLEELELFTDAVDRWDIRAMEQLPEYMKICYLALYNTTNDIAYEALKEEGLDVIPYLKKVWTDLCKSYIVEARWYSNGYKPTLEEYLENAWTSIAGPVALVHAYFSFGQKMPFEALNYSNTSSLIKWSSMIFRLCDDLATSSDEVARGDVPKSIQCYMYEAGVSESVARDHIKYLIDEAWKKMNECLVYNTPFLQPLINAGLNLARMAHCMYERGDGHGFSNELDKKRVLLLLAEPFKFM
